# Bias detection and correction in RNA-Sequencing data

**DOI:** 10.1186/1471-2105-12-290

**Published:** 2011-07-19

**Authors:** Wei Zheng, Lisa M Chung, Hongyu Zhao

**Affiliations:** 1 Biostatistics Resource, Keck Laboratory, Yale University, 300 George Street, New Haven, Connecticut, 06510, USA; 2 Biostatistics Division, Yale School of Public Health, 300 George Street, New Haven, Connecticut, 06510, USA

## Abstract

**Background:**

High throughput sequencing technology provides us unprecedented opportunities to study transcriptome dynamics. Compared to microarray-based gene expression profiling, RNA-Seq has many advantages, such as high resolution, low background, and ability to identify novel transcripts. Moreover, for genes with multiple isoforms, expression of each isoform may be estimated from RNA-Seq data. Despite these advantages, recent work revealed that base level read counts from RNA-Seq data may not be randomly distributed and can be affected by local nucleotide composition. It was not clear though how the base level read count bias may affect gene level expression estimates.

**Results:**

In this paper, by using five published RNA-Seq data sets from different biological sources and with different data preprocessing schemes, we showed that commonly used estimates of gene expression levels from RNA-Seq data, such as reads per kilobase of gene length per million reads (RPKM), are biased in terms of gene length, GC content and dinucleotide frequencies. We directly examined the biases at the gene-level, and proposed a simple generalized-additive-model based approach to correct different sources of biases simultaneously. Compared to previously proposed base level correction methods, our method reduces bias in gene-level expression estimates more effectively.

**Conclusions:**

Our method identifies and corrects different sources of biases in gene-level expression measures from RNA-Seq data, and provides more accurate estimates of gene expression levels from RNA-Seq. This method should prove useful in meta-analysis of gene expression levels using different platforms or experimental protocols.

## Background

Massive parallel sequencing of RNA has provided researchers a powerful tool for transcriptome analysis. The protocols of gene expression analysis using the sequencing approach fall into two major categories. The traditional approach is 3' tag digital gene expression (DGE), in which oligo-dT was used for synthesis of cDNA libraries, resulting in enrichment in the 3' end of polyadenylated mRNAs. These polyadenylated fragments were further digested by certain restriction endonucleases to produce short cDNA tags. The short tags were then sequenced by massive parallel sequencing technologies to give "digital counts" of the mRNA molecules originated from each gene. A variety of DGE approaches differ in their choices of restriction enonuclease, cDNA cloning step, and usage of barcodes [1,2]. These approaches were adapted from the original serial analysis of gene expression (SAGE) [3] and expressed sequence tag (EST) [4] approaches, and revolutionized by the ultra-high throughput brought upon by next-generation sequencing platforms. The more recent method is RNA-Seq, in which the entire transcriptome is randomly fragmented into pieces of a few hundred nucleotides, and reverse transcribed into cDNA libraries. These cDNA fragments are then PCR amplified and sequenced in parallel [5]. This technology requires considerably higher coverage to reach similar power in detecting lowly abundant transcripts. However, RNA-Seq provides more information than DGE on transcript structure and dynamics by surveying whole transcripts instead of 3' tags. With rapid reduction of sequencing cost and ever growing sequencing depth, RNA-Seq has gained more popularity over DGE approaches in recent years.

Compared to microarray-based gene expression analysis, DGE and RNA-Seq have essentially no background, and therefore overcome the ratio compression problem (the observed expression fold change is smaller than the real difference) persists in microarray technology; the dynamic range of sequencing based approaches also expands with higher sequencing depth [6]. Moreover, sequencing based gene expression measurement shows higher reproducibility between technical replicates than microarray based approaches [7,8]. Furthermore, it is possible to identify novel transcripts and isoforms with sequencing-based methods.

Despite these advantages, a comprehensive understanding of the characteristics and potential artefacts in the data is needed to most appropriately analyze RNA-Seq data. In an important study of RNA-Seq data characteristics, Oshlack et al. found transcript length biases in RNA-Seq data [9]. Specifically, the authors argued that due to the random RNA fragmentation and sampling nature in RNA-Seq, the aggregated read counts on a gene may follow a Poisson distribution, where the sampling rate is proportional to true expression level multiplied by gene length. Measures to normalize the aggregated read counts by gene length, such as reads per kilobase of exon model per million mapped reads (RPKM) [10] or similarly fragments per kilobase of exon model per million mapped reads (FPKM) [11], can potentially lead to unbiased estimate of the true expression level of a gene. However, the distribution of these normalized read counts no longer possesses the overall nice feature of equal mean and variance, and shorter genes have larger variance than longer ones. As a result, statistical power to detect differential expression will be a function of gene length. This phenomenon is inherent to the nature of RNA-Seq because there are essentially larger sample sizes for longer genes. On the other hand, DGE may not suffer from this issue because only one tag is counted for each gene. To overcome such gene length effect in detecting differentially expressed genes or enrichment of particular gene sets, a recent study proposed to use the square root of gene length as weights to adjust the gene level test statistic for differential expression, or to adjust the identification probability of each gene in the null distribution for the Fisher's exact test in gene set analysis [12].

In addition to length biases, other factors such as read mapping uncertainty and sequence base composition may also confound results from RNA-Seq experiments. Read mapping uncertainty across different isoforms [11] and different genes [13] have been addressed in rigorous statistical framework by recent methods in the field and improved estimation of gene expression levels from RNA-Seq experiment. In contrast, investigation of sequence base composition effect in RNA-Seq data is still exploratory [14,15]. The correlation between gene expression level and DNA base composition is an important and extensively studied issue. In mammalian genomes, the DNA base composition is correlated with gene density [16], recombination [17], methylation [18], and many other genomic features. But there is no clear evidence suggesting correlation between DNA base composition and gene expression pattern [19]. Base composition of microarray probes has been found to introduce variations to probe-target hybridization strength, and in turn affect the gene expression level measured by microarrays [20]. In high throughput DNA sequencing, it has been shown that various technical artefacts are correlated with base composition. Dohm et al. [21] clearly demonstrated a strong relationship between read counts and the GC content in 1 kb windows along the genome from Solexa DNA sequencing experiments. Recently, two groups independently examined the nucleotide-level read counts in RNA-Seq data and found strikingly similar non-uniform patterns in the reads distribution along genes; and the pattern is strongly associated with base compositions surrounding the investigated base position [14,15]. Hansen et al. [14] further showed that the pattern is specific to RNA-Seq libraries made by random hexamer priming, and proposed a random hexamer based re-weighting approach to adjust for the nucleotide composition bias in read counts. Alternatively, Li et al. [15] regressed the read counts at a particular base on the identities of nucleotides around the base, using parametric and non-parametric regression tools. Besides random hexamer priming efficiency, the base composition may affect RNA secondary structure or priming efficiency in other enzymatic steps, and lead to uneven sequence read coverage from the cDNA sequencing library. We expect that the base-level composition bias may also affect our estimation of gene expression levels. Overlooking such biases may mislead downstream biological interpretations.

In this article, based on multiple publicly available RNA-Seq data sets, we showed that the commonly used measures of gene expression levels, such as raw read counts or RPKM/FPKM, are biased in transcript length, GC content, and dinucleotide frequencies. We also investigated the effects of biological sources, experimental protocols and data processing methods on these bias patterns systematically. We found that the bias patterns are specific to experimental protocols but not specific to biological sources, suggesting that they are technical artefact rather than biologically relevant patterns. It motivated us to develop a generalized additive model based algorithm to jointly correct for these biases. In contrast to [14] and [15] where base-level read counts were investigated, we focused on aggregated read counts for each gene and aim to remove the bias trend across genes. Our method can effectively correct for gene-level biases, and the corrected estimates of absolute gene expression levels agree better with gold-standard expression measures such as Taqman RT-PCR and QuantiGene. Compared to the random hexamer bias correction method in [14] and multiple additive regression trees (MART) in [15], our method is computationally less demanding, yet performs more favourably in correcting for gene-level biases. Our work not only contributes to understanding of potential technical artefacts in RNA-Seq experiments, but also provides more accurate gene expression measures. The correction method will be useful in meta-analysis of data sets using different platforms or experimental protocols, and may also help correcting for tissue/cell-type heterogeneity in RNA samples. Obtaining accurate absolute gene expression levels is also of inherent biological interest, and help answering various biological questions, such as estimating enzyme reaction rates for genetic network modelling, comparison of isoform levels, and evolutionary comparisons across species.

## Methods

### Data sets

#### MAQC data sets

MicroArray quality control (MAQC) data sets [22,23] contain gene expression data from multiple quantitative platforms and are widely used in assessing platform performance and testing for different data processing methods. We used data collected through three platforms for two samples: the Ambion Human Brain Reference RNA and Stratagene Universal Human Reference RNA. The three platforms are Solexa 1 G Genome Analyzer sequencing platform [22], Taqman qRT-PCR platform, and QuantiGene platform [23].

The two samples are labelled MAQC2 and MAQC3. MAQC2 contains 7 technical replicates of brain reference RNA samples and 7 technical replicates of UHR RNA samples sequenced on 14 lanes of two flow cells. MAQC3 contains UHR RNA samples from 4 different library preparations sequenced on 14 lanes of two flow cells.

All experiments used standard Illumina RNA-Seq protocol. The raw sequence files were downloaded from the Sequence Read Archive http://www.ncbi.nlm.nih.gov/sra/ with accession numbers SRX016366, SRX016368-SRX016372. Taqman quantitative RT-PCR technology first reverse transcribes RNA transcripts into cDNA, and then measures concentration of each cDNA template at each cycle of PCR by detecting the fluorescent signals released from a target-specific fluorogenic hybridization probe that is hydrolyzed by Taq polymerase during the extension phase. It is often used as gold standard for microarray studies. Taqman qRT-PCR data for 1,044 probes, corresponding to 1,001 RefSeq genes, were obtained from GEO database with accession numbers GSM129638-GSM129645, which represent 4 technical replicates of Taqman qRT-PCR for each of the two samples. Reported values are normalized expression values as . The expression of each measured gene was compared to the housekeeping gene POLR2A by delta CT (cycle threshold) calculations, a CT value of 35 (detection threshold) was used for any replicate that had CT > 35. QuantiGene is a highly sensitive assay based on DNA-RNA hybridization and does not involve any reverse transcription step. The amount of RNA molecules is measured by luminescence signals released during branched DNA amplification. The detection threshold of QuantiGene data is background + 3 SD of background. Background signals were determined in the absence of RNA samples and subtracted from signals obtained in the presence of RNA samples. QuantiGene data for 244 RefSeq genes were downloaded from GEO database with accession numbers GSM129654-GSM129659, representing 3 technical replicates for each of the two samples. Reported values are background-subtracted luminescence signal. Both Taqman and QuantiGene data sets labelled genes above detection threshold with a flag "P" and those below the threshold with "A", and we only used genes with flag "P" in downstream analysis. To compare with RNA-Seq data, we matched RefSeq gene IDs with ENSEMBL gene IDs (v59) using BioConductor, and only used genes with unambiguous one to one match in downstream analysis.

#### RNA-Seq data from kidney and liver

This dataset was generated in [7] and used widely in the literature on RNA-Seq data analysis approaches. Raw sequence files from liver (SRX000571) and kidney (SRX000605) were downloaded from the Sequence Reads Archive. To compare with the data processing method used in the original publication, read counts for each Ensembl gene were downloaded from http://bioinf.wehi.edu.au/resources/ [24].

#### Yeast RNA-Seq data

This dataset was described in [25] and also used in [14] to demonstrate sequencing biases caused by random hexamer priming. We downloaded raw sequence files of two technical replicates of the isogenic wild-type strain from the Sequence Read Archive with accession numbers SRR014335 and SRR014336.

#### Yeast RNA-Seq data using alternative protocols

This dataset was described in [26] through different library preparation procedures. Standard RNA-Seq library preparation involves RNA fragmentation followed by cDNA synthesis with random hexamer priming. In this study, cDNA synthesis before fragmentation and different priming chices was tested. We downloaded raw sequence files of two libraries prepared by random hexamer priming and cDNA fragmentation by DNase I (SRR002058), and oligo-dT priming and cDNA fragmentation by DNase I (SRR002062) from the Sequence Reads Archive.

#### FRT-Seq data

This dataset was generated in [27] for human placental poly(A)+ RNA sample, using both the standard RNA-Seq library preparation protocol and a novel approach called FRT-Seq. This new approach does not involve PCR amplification and the reverse transcription step is performed right on flow cell (FRT). We downloaded the raw sequence files for FRT-Seq (ERR007689) and standard RNA-Seq (ERR007710) from the European Nucleotide Archive. To compare with data processing method used in the original publication, read counts for each RefSeq gene were also downloaded from ftp://ftp.sanger.ac.uk/pub/transseq/.

**Figure 1:**
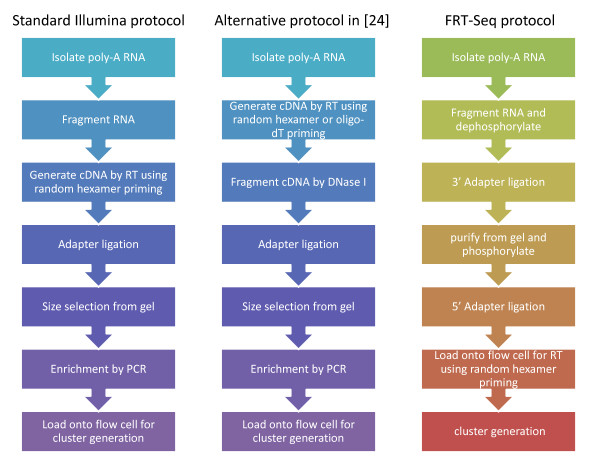
**Differences in experiment protocols for RNA-Seq**. Major steps in standard Illumina RNA-Seq sample preparation protocol in Marioni et al. [7], Bullard et al. [20], Lee et al. [23] (left), alternative RNA-Seq protocols in Nagalakshmi et al. [24] (middle) and FRT-Seq protocol in Mamanova et al. [25] (right) are compared.

All the data sources, accession numbers, characteristics of the techniques and related information are listed in Table 1. The differences between sample preparation protocols in these data sets are summarized in Figure 1.

**Table 1:** Summary of RNA-Seq Data sets used in this study.

Data Set	Source	Accession Number	Technology Characteristics	Notes	References
MAQC data set	Sequence Read Archive	SRX016366, SRX016368-SRX016372	Standard Illumina RNA-Seq protocol: RNA fragmentation, random hexamer priming	expression data also available from Taqman RT-PCR and QuantiGene platforms	[22]

RNA-Seq data from kidney and liver	Sequence Read Archive	SRX000571 SRX000605	Standard Illumina RNA-Seq protocol: RNA fragmentation, random hexamer priming	read counts per gene available from http://bioinf.wehi.edu.au/resources/	[7]

Yeast RNA-Seq data	Sequence Read Archive	SRR014335 SRR014336	Standard Illumina RNA-Seq protocol: RNA fragmentation, random hexamer priming	NA	[25]

Yeast RNA-Seq data using alternative protocols	Sequence Read Archive	SRR002058 SRR002062	random hexamer or oligo-dT priming; cDNA fragmentation by DNase I	NA	[26]

FRT-Seq data	European Nucleotide Archive	ERR007689 ERR007710	standard protocol or FRT-Seq (no PCR, RT step on flow cell)	read counts per gene available from ftp://ftp.sanger.ac.uk/pub/transseq/	[27]

### Data processing procedure

Three procedures were performed in processing sequence data to obtain estimates of expression levels. For Procedure 1, we used TopHat (v1.1.0) [28] to align the raw sequencing reads to reference genome, using gene model annotations to direct the mapping of reads spanning splice junctions. In this default setting, reads that can be mapped to multiple (less than 10) genomic locations had all alignments reported and their contributions in estimating gene expression were proportionally down-weighted by Cufflinks. The specific reference genome and gene model annotation used for each data set are listed in Additional file 1. Cufflinks (v0.9.3) [11] was then used to estimate gene expression levels based on the same gene model annotations. By specifying the known gene structure annotations, we suppressed Cufflinks from de novo transcript assembly, and only allowed it to assign reads to different isoforms probabilistically, estimate isoform-level expression using maximum likelihood method similar to rSeq [29], and summarize isoform-level expression into gene-level expression measures in FPKM unit. The gene expression levels estimated from Cufflinks agreed well with those from rSeq (results not shown). For Procedure 2, we directly extracted read counts per gene or RPKM from original publications if they are available and compared them with corresponding gene model annotations. Due to the limited availability of intermediate data and ambiguity of originally used gene model annotations, we only implemented this procedure for Marioni et al. liver and kidney data set and Mamanova et al. FRT-Seq data set. For Procedure 3, we directly counted reads whose starting positions fall in exon regions of all transcripts from mapping results obtained in Procedure 1, and converted them to RPKM units. The procedures applied to each data set are listed in Additional file 1.

### Bias corrections by generalized additive models

We consider the bias of gene expression levels in terms of gene length, gene GC content, and the frequencies of the 16 possible dinucleotides. Let *Y_i _*be the inferred gene expression level for gene *i *(from one of the three procedures discussed above), and ***X ***be a matrix with the *i*-th row *X_i _*≡ (*X*_*i*, 1_, *X*_*i*, 2_,..., *X*_*i*, 18_) representing the values of 18 potential bias factors for gene *i*. Specifically, *X*_*i*, 1 _is the gene length on log scale, and (*X*_*i*,2_,..., *X*_*i*, 18_) are bias factors related to nucleotide composition. Our goal is to obtain a less biased estimate of expression level  by removing the biases due to these factors. Since some of the bias factors are correlated (e.g. GC content is correlated with GC dinucleotide frequencies), we first performed a principal component analysis on the 2nd to 18th columns of ***X***, denoted as ***X***_(-1)_, and obtain the first K principal components *P*_*i*, 1_, *P*_*i*, 2_,..., *P*_*i*, K _that can explain more than 95% variation in ***X***_(-1)_. Then we used these principal components to fit the following generalized additive model (GAM): *log*(*Y*_*i*_) = *α*_*i *_+ *X*_*i*, 1 _+ *s*(*P*_*i*1_) + *s*(*P*_*i*2_) +⋯+ *s*(*P*_*iK*_) + *ε*_*i*_. Finally we obtain the bias corrected expression as the sum of the grand mean of *Y *and the residual from fitted model, i.e. .

### Definition of "Gene" units to summarize expression levels

In mammalian genomes, one gene may contain multiple transcript isoforms, and sequencing reads can fall on a genomic region corresponding to different isoforms or even genes. What is more, there is no consensus on "gene" unit to summarize gene expression. For example, Oshlack et al. defined gene length as the median of all transcript length related to that gene, which is a simple high level summary but may not be accurate when different isoforms have very different expression levels [9]. Hansen et al. defined region of constant expression (ROCE) by dividing genomic regions based on which gene and transcript isoform(s) the regions are associated with [14]. This definition avoids the ambiguity in assigning sequence reads to different transcript isoforms, but loses the biological interpretation for each unit. Li et al. avoided ambiguity in overlapping genes/isoforms by focusing on single isoform non-overlapping genes [15]. This definition inevitably loses information from the data. In this study, we examined different units (transcripts, genes, and SINO genes) to summarize expression level and detect biases, and therefore alleviate the potential artifact associated with each definition. We used the median cDNA length, cDNA GC content and dinucleotide frequencies as gene-level summaries.

### Software package

The GAM correction method and facilitating functions were implemented in an R package (RNASeqBias) and this package can be obtained as Additional file 2, and also from http://bioinformatics.med.yale.edu/group/. This GAM-based correction method is computationally efficient. It takes ~4-5 seconds on a 2.67 GHz Xeon CPU to correct ~30,000 gene expression levels for human genome with ENS 59 annotation.

## Results

### RNA-Seq biases in length, GC content, dinucleotide frequencies

**Figure 2:**
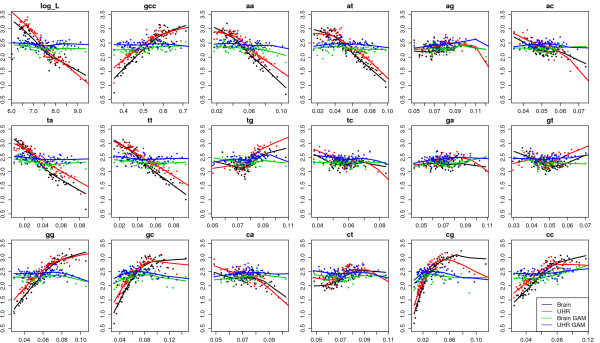
**Bias plots for MAQC data (Procedure 1, gene-level)**. Genes were grouped into bins according to log gene length, GC content, and dinucleotide frequencies, and the median expression levels in log(FPKM) units versus median bias factors were plotted for MAQC2 brain and UHR samples before and after GAM correction. Each bin contains 500 genes. Data were processed by Procedure 1. This data set showed strong linear relationship between expression levels and gene length, GC content and dinucleotide frequencies that are related to GC content (i.e. AA, AT, TA, TT, GG, GC, CG, CC). Moreover, the patterns from two different biological samples (brain and UHR) were very similar. After GAM correction, the bias patterns diminished.

To detect potential biases in the gene expression levels, we grouped genes into bins according to each of the potential bias factors, e.g. gene length, GC content, and dinucleotide frequencies, and plotted the median expression levels versus median bias factors for the five data sets in Figure 2 and Additional file 3. MAQC data set showed strong linear relationship between expression levels and gene length, GC content and dinucleotide frequencies that are related to GC content (i.e. AA, AT, TA, TT, GG, GC, CG, CC). Moreover, the patterns from two different biological samples (brain and UHR) were very similar. In the Marioni data set, the expression levels showed negative linear correlation with gene length and CA dinucleotide frequency, and mostly quadratic patterns for the other nucleotide composition variables. Again, the patterns from two different biological samples (liver and kidney) were very similar. The Mamanova data set showed similar patterns as the Marioni data set, with mostly quadratic patterns and negative linear correlation between expression and gene length. The patterns for FRT-Seq and STD-Seq are different mainly in CA and AC dinucleotide frequencies. The two yeast studies showed similar linear patterns although different library preparation protocols were used. In both studies, expression level had negative correlation with gene length, AT, TA, positive correlation with GC content, TG, GT, GG, GC, CC dinucleotide frequencies. In summary, there are clear non-random patterns between estimated expression levels and gene length and nucleotide composition, and the patterns were not specific to the biological samples being sequenced, which indicated that the patterns were caused by technical issues rather than biological features.

Many data processing tools have been developed for RNA-Seq data analysis in the literature. To evaluate whether the bias pattern we observed in RNA-Seq data is due to certain data processing methods involved in the processing of these data, we implemented multiple processing procedures for each data set, and summarized expression levels using different "gene" unit definitions (see Methods section). Procedure 3 differs from Procedure 1 in that reads will be double counted instead of being assigned to different isoforms probabilistically for overlapping transcripts or genes. Even for single isoform non-overlapping (SINO) genes, Procedure 1 may be different from Procedure 3 due to two features of Cufflinks. First, when gene model annotation (.gtf) file is provided, Cufflinks only counts fragments that fall on gene model features for the "total number of fragments mapped". Second, Cufflinks uses each fragment's map quality to down-weight reads that were mapped to multiple genomic locations. By comparing bias patterns from these different procedures, we examined whether different mapping policies, gene model annotations and expression estimation methods have any effect on the observed bias patterns. We found that all three data processing methods and both gene level and transcript level expression estimates yielded similar patterns (Additional files 4 and 5). However, the patterns were less obvious when we focused only on expression estimates of SINO genes, probably for the reason that the proportion of SINO genes is low in protein coding genes (Additional file 6). Out of 49,733 annotated Ensembl 59 chromosomal genes, there are 20,555 SINO genes; out of 21,727 protein coding genes, there are only 3,870 SINO genes. Since most RNA-Seq experiments enriched for poly(A)^+ ^mRNAs transcribed from protein coding genes, investigation using only SINO genes may lose considerable amount of information, and patterns learned from SINO genes may not be applicable to general protein coding genes.

### Relationship between gene-level biases and base-level biases

In the previous section, we examined bias patterns between estimated expression level and nucleotide composition across genes. To examine bias patterns between base level read counts and nucleotide composition, we compared the percentage of dinucleotides at the starting position of all mapped reads from different data sets (Table 2). Mamanova et al. sequenced the same biological sample using two different protocols, and clearly showed protocol-specific biases. STD-Seq protocol was also used in data sets from Marioni et al., Bullard et al, and Lee et al., and these data sets all showed significant over-representation of dinucleotides starting with C and G, but the extent of over-representation depended on different studies. Nagalakshmi et al. used two different types of primers (oligo-dT or random hexamer) in cDNA synthesis but they showed similar patterns, which were different from those using STD-Seq protocols, indicating that choices of primers were not the main cause of the bias patterns in this study. Instead, the library preparation step that is common in the Nagalakshmi study but different from STD-Seq caused the biases, i.e. fragmentation of cDNA instead of RNA.

**Table 2:** Percentage of dinucleotide at the starting position of all mapped reads.

		**Mamanova et al**.		**Marioni et al**.		**Bullard et al**.			Lee et al	**Nagalakshmi et al**.	
**Dinuc**	**Read type**	**STD-Seq**	**FRT- Seq**	**Marioni kidney**	**Marioni liver**	**MAQC2 brain**	**MAQC2 UHR**	**MAQC3 UHR**	**yeast RH**	**yeast oligodT**	**yeast RH**

AA	unique	4.5	**9.3**	6.1	**8.4**	2.1	2.2	2.7	5.2	**10.8**	**10.3**
AA	multi	4.5	**10.3**	6.7	7.9	2.5	2.6	3.0	4.0	**10.1**	7.9
AC	unique	1.5	**5.2**	1.4	1.4	0.9	1.0	0.8	1.4	6.4	5.4
AC	multi	2.1	**5.8**	1.3	1.3	1.0	1.0	0.9	1.3	6.0	5.2
AG	unique	2.5	**7.1**	3.0	3.2	2.2	2.2	2.2	2.3	**7.8**	5.9
AG	multi	3.3	6.6	2.8	3.0	2.1	2.2	2.1	2.0	**6.9**	4.8
AT	unique	3.4	**7.2**	5.7	**6.8**	1.9	2.0	2.4	4.7	7.4	5.7
AT	multi	3.9	5.4	6.0	**7.0**	2.3	2.4	2.7	4.3	7.8	5.0

CA	unique	**8.9**	5.4	**7.7**	**7.0**	**10.7**	**10.6**	**10.5**	**8.7**	**10.6**	**11.1**
CA	multi	7.3	5.2	6.2	5.6	**10.0**	**11.0**	**10.9**	**7.8**	**10.9**	**10.2**
CC	unique	**9.6**	4.0	**7.8**	5.8	**10.8**	**10.2**	**10.4**	5.6	**6.0**	**6.3**
CC	multi	**9.0**	4.2	**7.2**	5.4	**9.6**	**8.8**	**9.4**	**7.3**	**5.5**	**7.7**
CG	unique	**5.5**	2.3	**4.4**	**3.4**	**10.0**	**10.1**	**10.4**	**7.2**	2.0	2.9
CG	multi	**5.8**	2.9	**5.2**	**4.6**	**9.9**	**8.7**	**9.1**	**8.6**	2.2	**4.3**
CT	unique	**12.2**	5.8	**13.3**	**11.6**	**13.1**	**13.0**	**13.3**	**12.2**	**7.9**	**7.4**
CT	multi	**13.0**	6.0	**13.0**	**11.2**	**13.4**	**13.5**	**13.7**	**13.1**	**7.8**	**9.1**

GA	unique	4.0	**6.7**	4.9	5.3	5.2	5.4	4.5	**7.0**	4.9	5.4
GA	multi	4.5	6.1	4.7	5.2	5.3	5.8	4.7	6.2	5.0	5.3
GC	unique	**12.5**	5.7	**7.7**	**6.9**	**11.7**	**12.0**	**10.0**	**8.6**	2.2	3.0
GC	multi	**11.2**	**6.2**	**8.1**	**7.6**	**11.1**	**11.6**	**9.8**	**10.3**	1.8	3.2
GG	unique	**13.0**	5.0	**9.2**	**8.8**	**12.0**	**11.9**	**11.8**	**8.7**	3.8	**4.2**
GG	multi	**11.7**	6.0	**9.8**	**9.6**	**11.7**	**11.4**	**11.1**	**8.6**	**4.1**	**5.2**
GT	unique	**9.8**	**5.7**	**10.1**	**9.5**	**8.4**	**8.6**	**8.4**	**12.3**	4.2	5.2
GT	multi	**9.2**	5.0	**10.4**	**10.0**	**9.2**	**9.1**	**9.1**	**11.8**	4.6	**6.9**

TA	unique	1.6	**5.2**	3.7	4.4	1.1	1.1	1.3	2.2	**10.8**	**10.8**
TA	multi	1.7	3.8	4.3	5.0	1.4	1.1	1.4	2.1	**11.2**	**10.3**
TC	unique	2.1	**6.8**	3.0	3.2	2.1	2.2	2.3	2.9	3.9	4.8
TC	multi	2.8	6.0	2.7	3.0	2.1	2.2	2.3	3.0	3.8	4.2
TG	unique	6.1	6.9	5.6	5.6	4.7	4.9	5.4	5.1	**7.9**	**8.4**
TG	multi	5.7	**8.2**	5.5	5.6	4.8	5.3	5.8	4.7	**8.7**	**7.8**
TT	unique	2.7	**11.4**	6.3	8.4	2.5	2.5	3.6	5.9	3.5	3.3
TT	multi	4.1	**12.1**	6.2	7.9	2.9	2.9	4.0	4.8	3.4	2.7

Percentage was calculated by SAMStat [33]. Uniquely mapped reads and multi-mapped reads were separated in the calculation but showed similar pattern. Bold numbers indicates significantly over-represented dinucleotide with p-values less than or equal to 10^-100 ^from a Binomial test described in [34].

The base-level and gene-level bias patterns from the same study (except for the Mamanova data) tended to have similar patterns although different biological samples or library preparation protocols were used, which indicates experiment-specific biases. In the MAQC data set, both base-level and gene-level patterns indicate strong GC content related bias. However, they do not always agree well, and probably capture different characteristics of the data. For example, there are strong base-level biases for reads starting from CT and GT, but this is not reflected in the gene-level bias patterns.

### Generalized Additive Model to correct for gene-level biases

**Figure 3:**
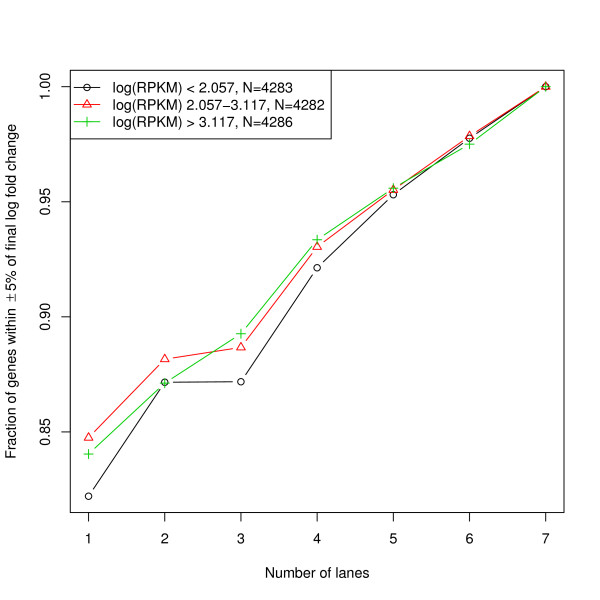
**GAM is robust to sequencing depth and gene expression levels**. Seven lanes of MAQC2 UHR data to perform GAM correction by adding one lane at a time. The log fold changes of estimated gene expression levels before and after GAM correction were calculated for genes with high, medium and low expression. Fractions of genes with log fold change within ± 5% of the final value were plotted. Overall the correction was robust to sequencing depth, with ~80% genes showing the fold change within ± 5% of the final estimates using only one lane. Moreover, genes with lower expression were only slightly more sensitive to sequencing depth.

To correct for the observed gene-level biases, we calculated principal components of dinucleotide compositions and gene length, and fitted a generalized additive model of log(FPKM) on the eigenvectors (see Methods section). As shown in the bias plots (Figure 2 and Additional files 3, 4, 5 and 6), GAM can effectively reduce the correlation between gene expression and gene length or nucleotide compositions. We also found that GAM correction is robust to sequencing depth and corrected gene expression level estimation. We used seven lanes of MAQC2 UHR data to perform GAM correction by adding one lane at a time. The log fold change of estimated gene expression levels before and after GAM correction was calculated for genes with high, medium and low expression. Fractions of genes with log fold change within ± 5% of the final value were plotted (Figure 3). Overall the correction is robust to sequencing depth, with ~80% genes showing the fold change within ± 5% of the final estimates using only one lane. Moreover, genes with lower expression were slightly more sensitive to sequencing depth.

### Comparing RNA Sequencing data with other quantitative platforms

We compared the expression estimates from RNA-Seq data with Taqman RT-PCR and QuantiGene platforms, which measure gene expression levels using different technologies. The purpose is two-fold. First, by comparing the patterns of expression levels across genes from different platforms, we could distinguish technical bias from biological patterns. Technical bias should be specific to certain platforms, whereas biological patterns should be consistent across platforms. Second, by comparing the estimated expression levels for each gene from different platforms, we could evaluate whether the GAM correction is able to reduce technical biases. If the corrected expression estimates from RNA-Seq data are less biased, we would expect it to correlate better with other quantitative platforms.

**Figure 4:**
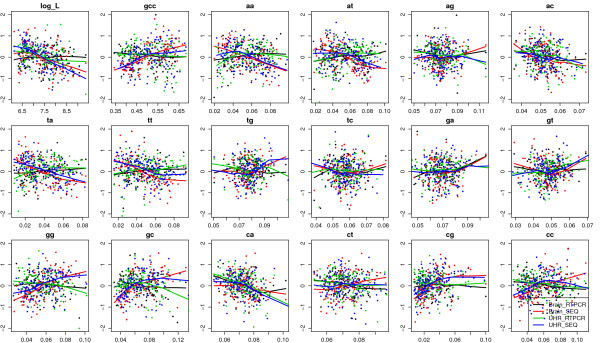
**Platform and sample specific biases**. Bias patterns of gene expression levels in terms of log gene length, GC content and dinucleotide frequencies for 571 and 606 genes measured by both Taqman RT-PCR (expression levels in ΔCT unit) and RNA-Seq platforms (expression levels in log FPKM unit) and expressed above the detection thresholds in brain and UHR samples. The expression levels were rescaled to mean of 0 and standard deviation of 1 on Y-axes. In most bias plots, the fitted lowess curves for sequencing platform (blue and red) were separated from those from RT-PCR platform (green and black), which indicates platform specific biases. Only a few plots (e.g. for AG and GA dinucleotide frequencies) showed separation between brain samples and UHR samples, which indicates sample specific biases.

We compared the patterns of gene expression levels in terms of gene length, GC content and dinucleotide frequencies for 571 and 606 genes measured by both Taqman RT-PCR and RNA-Seq platforms and expressed above the detection thresholds in brain and UHR samples (Figure 4). Similarly, we compared 165 and 184 genes that were measured by both QuantiGene assay and RNA-Seq platforms and expressed above the detection thresholds in brain and UHR samples. A linear model was built to regress expression levels (in log(FPKM) unit) on each variable (gene length, GC, or dinucleotide frequencies), including an indicator for platforms (Seq vs. RT-PCR) and an indicator for samples (brain vs. UHR). Then we tested whether the indicator terms affected the linear relationship between expression and each factor by t-tests and reported the p-values (Additional file 7). We found that the linear pattern between expression and gene length together with all dinucleotide frequencies were significantly different between RNA-Seq and RT-PCR platforms, and pattern with GC content, AA, AT, TA, TT, GG, GC, CG, CC dinucleotide frequencies were significantly different between RNA-Seq and QuantiGene platforms. On the other hand, all but one comparison between brain and UHR samples were not significantly different. The linear relationship between expression levels and gene length was significantly different between RNA-Seq and RT-PCR platforms, but only marginally significant between brain and UHR samples. There are fewer significant terms in RNA-Seq vs. QuantiGene platform comparison than those in the RNA-Seq vs. RT-PCR platform comparison. One possible reason is that there are fewer genes in the former comparison. Nonetheless the significant terms were all related to GC content, consistent with the most significant biases in Figure 2.

To examine the relationship between expression measurements from different platforms, we performed a series of pair-wise comparisons through Pearson's correlations. Multiple technical replicates were averaged for each platform, and the units for expression levels were ΔCT for RT-PCR, log(luminescence signal) for QuantiGene, and log(FPKM) for RNA-Seq. We first considered 182 and 200 genes that were measured by both Taqman RT-PCR and QuantiGene assays and expressed above the detection thresholds in brain and UHR samples respectively. The correlation was 0.810 for brain sample, and 0.815 for UHR sample. Then we compared genes present on both Taqman RT-PCR and RNA-Seq platforms. The correlation coefficients (r) were 0.690 (brain) and 0.748 (UHR) before GAM correction, and increased to 0.754 (brain) and 0.763 (UHR) after GAM correction. Furthermore, when we stratified genes into three quartiles by the absolute value of log fold change between corrected and uncorrected expression levels, the relative improvement in correlation increased as correction magnitude increased (Table 3). Similar results were obtained when we compared genes that were present on both RNA-Seq and QuantiGene platforms (Table 3). The improvement trend is consistent but less obvious in UHR sample, probably because UHR sample is a mixture of ten human tissue cell lines with heterogeneous splicing patterns. These results suggested that GAM correction for RNA-Seq data may improve the consistency in gene expression estimates across quantitative platforms.

**Table 3:** Comparison of correlations (*r*) between MAQC2 RNA-Seq data and other platforms.

Platform1	Platform2	Sample	Log fold changes	Original *r*	*r *after GAM Correction	%Relative improvement
RNA-Seq	Taqman	Brain	(0, 0.326]	0.749	0.747	-0.3
			(0.326, 0.731]	0.664	0.699	5.3
			(0.731, 2.1]	0.655	0.807	23.2
			**Overall**	**0.69**	**0.754**	**9.3**

RNA-Seq	Taqman	UHR	(0, 0.368]	0.778	0.774	-0.5
			(0.368, 0.753]	0.776	0.79	1.8
			(0.753, 1.78]	0.704	0.724	2.8
			**Overall**	**0.748**	**0.763**	**2**

RNA-Seq	QuantiGene	Brain	(0, 0.3]	0.833	0.823	-1.2
			(0.3, 0.743]	0.659	0.721	9.4
			(0.743, 1.69]	0.647	0.738	14.1
			**Overall**	**0.725**	**0.766**	**5.7**

RNA-Seq	QuantiGene	UHR	(0, 0.375]	0.832	0.839	0.8
			(0.375, 0.793]	0.811	0.759	-6.4
			(0.793, 1.78]	0.632	0.692	9.5
			**Overall**	**0.771**	**0.777**	**0.8**

Gene expression levels from Taqman RT-PCR or QuantiGene platforms are compared with RNA-Seq data, before and after bias correction by GAM.

### Relative contribution of each component in the GAM model

To understand the correlation structure between individual bias factors and the principal components going into the GAM model, we plotted the principal components found in 8 data sets covering all the sample organisms, tissues, library prep procedures, and data processing procedures (Additional file 8). Procedures 1 and 3 do not differ in the principal component analysis step, so we only showed examples from procedures 1 and 2. Features presented in these plots include the variance explained by each principal component, the minimal number of principal components that explain at least 95% of the variance, and the biplots [30] showing the variance, covariance and correlations among predictors, and the components of the first two principal components. The GC content shows strong positive correlation with dinucleotide frequencies of GC, CG, GG, and CC, and negative correlation with dinucleotide frequencies of TA, AT, TT, and AA. These predictors are the major drivers of the first principal component in MAQC, Marioni and FRT-Seq data sets, whereas yeast data sets (Lee and Nagalakshmi) exhibit somewhat different correlation patterns. There are no strong correlations between gene length and GC in any of these data sets, as the absolute values of r are all less than 0.1 (data not shown). These observations suggest that the GC content is not the solely important factor in explaining the variation in our design matrix.

To investigate how many of these components are actually important in our model, we calculated the nonparametric F-statistic and corresponding p-value for each predictor (gene length and PCs) from the fitted generalized additive models for the same 8 examples as above (inset tables in Additional file 8). Gene length and the first principal component are almost always highly significant, and additional principal components are also significant in some examples. In addition, for data sets with similar correlation structures among predictors, such as MAQC brain and MAQC UHR data sets, the marginal significance of each PC is different, indicating that gene length and GC content (mostly driving the first PC) alone may fail to capture the bias patterns in the estimated gene expression levels in some cases.

We also investigated the effect of including different number of principal components in the GAM model on the cross-platform correlations in MAQC2 data sets with RNA-Seq, RT-PCR and QuantiGene platforms. In these cases, correction using gene length and the first principal component in general shows the best performance. Including more principal components has little effect on cross-platform correlations (Additional file 9). However, we note that this may not be a general phenomenon for other data sets. As shown in Table 2 MAQC data sets have a distinct pattern that reads starting with C or G are highly enriched whereas no other dinucleotide distribution bias was detected. This is not the case in other data sets such as Lee, Nagalakshmi and Mammanova FRT-Seq data sets. Unfortunately due to the lack of information from "gold-standard" platforms, we cannot directly assess the improvement in accuracy in these data sets.

To explore the correction effect of using GAMs, we calculated the correlation between corrected gene expression levels using only length and GC, and corrected gene expression levels using length plus 1, 2, 3 or minimal number of principal components explaining 95% of variance (Additional file 10). For all 8 data sets tested, the correlations are very high (r > 0.84), indicating that at least for these data sets, the correction is insensitive to the number of principal components included in GAM, and that their performances are similar to the correction using only gene length and GC content in GAM. On the other hand, the GC bias patterns observed in the original data and corrected by GAM are actually very different across 8 data sets (Additional file 11). For example, the Marioni data sets and Mamanova data sets showed quadratic bias patterns in GC content, MAQC data sets showed almost linear bias patterns in GC content, Lee data sets and Nagalakshmi data sets showed non-linear bias patterns in GC content; and these different forms of bias patterns can all be corrected by the GAM model, indicating that GAM is flexible enough to detect and correct for different bias patterns specific to each experiment. This property is particularly useful when we try to apply a general correction method to sequencing data from different experimenters or protocols that may carry different technical biases.

### Comparison with other methods

We compared the GAM correction method with two other methods correcting for base-level nucleotide composition biases. We first implemented the random hexamer bias correction method proposed by Hansen et al. using the R package Genominator [14]. Sine this method corrected read counts at each base position along the gene according to nucleotide composition at the first several nucleotides along the mapped reads, we compared it with gene expression estimated from Procedure 3. The corrected base level counts were summed over each gene, and divided by the sum of weights to represent gene expression levels comparable to RPKM units. From the bias plots we can see that the random hexamer bias corrected gene expression levels did not change the original levels much, and still showed the same bias patterns (Additional files 12, 13).

We also implemented the regression methods proposed by Li et al. using the R package mseq [15]. Since this method was designed for SINO genes, and required extensive computation time, we only tested it on the yeast data set from Lee et al. The most highly expressed 100 SINO genes were used as training set and all other genes were used as testing set. Forty nucleotides were trimmed from both ends of genes, and nucleotide compositions in 40 upstream and downstream bases around the starting position of each mapped read were included in the Poisson linear model. The coefficients were larger at positions closer to the read starting position as expected, but the cross validated R^2 ^was only 0.086. Based on the coefficient plot, we chose 15 upstream nucleotides and 30 downstream nucleotides to fit a MART model. The cross-validated R^2 ^for MART model was 0.107. We then obtained gene-level expression estimates by dividing the sum of read counts over the gene with the sum of MART fitted sequencing preferences at each position of the gene (trimmed regions were not considered). Similar to the random hexamer bias correction method, the mseq-corrected gene expression levels were very close to uncorrected expression levels, and yielded the same bias patterns across genes (Additional file 13).

We further compared the reweighted expression levels with measurements from the Taqman RT-PCR and QuantiGene platforms. We first confirmed that the gene expression levels calculated using Procedure 3 agreed better with RT-PCR after GAM correction by increasing the Pearson's correlation from 0.793 to 0.817 for brain sample, which is consistent with the observation made from Procedure 1. We then compared the gene expression levels calculated using Procedure 3 before and after the random hexamer bias correction, and calculated their correlation with RT-PCR measured gene expression. The random hexamer bias correction method didn't improve the cross-platform correlations. However, when we applied GAM correction on random hexamer bias corrected expression levels, the correlation with RT-PCR data improved from 0.794 to 0.815 (Additional file 14). These results suggested that the bias patterns across genes were not adequately addressed by the base-level bias correction methods, and GAM correction is necessary to further remove these biases.

## Discussion

In this article, we examined the gene-level expression estimates from several published RNA-Seq data sets, and found systematic biases related to nucleotide composition and gene length. We proposed a generalized-additive-model based method to correct for these biases and achieved better cross-platform comparison results.

The major difference between our method and previous studies exploring the biases in RNA-Seq data is that we summarized and corrected for the biases at the gene level instead of the base level. Previous studies (e.g. Li et al. 2010 and Hansen et al., 2010) employed parametric or non-parametric regression techniques to relate base-level read counts to local nucleotide composition. While achieving better uniformity in read distribution within genes, these methods only corrected for gene level expression estimation to a limited extent, as shown in Additional files 12 and 13. Moreover, the biases detected on gene level and base level are likely to be complementary to each other. For example, the FRT-Seq data from Mamanova et al. showed gene-level biases that were different from standard sequencing data (Additional file 3), whereas no base level biases in nucleotide compositions were detected in Hansen et al. [14]. In fact, combining GAM correction for gene level biases and random hexamer bias correction scheme for base level biases performed better than random hexamer bias correction alone in cross-platform comparison of gene expression measures (Additional file 14).

During the review of our manuscript, a related correction method for RNA-Seq data was published [31]. This method was built on top of the maximum likelihood based algorithm for isoform/gene expression level estimation in Cufflinks [11], and modified the original likelihood function by introducing 100 weights parameters that measure positional biases. The estimation of the relative transcript abundance and bias parameters can be jointly estimated using an iterative coordinate ascent procedure, although in practice only one iteration was used. This method focuses on adjusting the base-level positional biases similar as [15], and therefore complements our focus on gene-level bias correction. In another newly published eQTL study with RNA-Seq platform [32], the authors observed GC bias on sequencing depth across different sequencing lanes, and proposed a direct correction of GC content by fitting a spline to the plot of log_2 _relative enrichment of exon level read counts in each GC bin against the mean GC content for the bin. The binning and curve fitting strategy is similar to our method but it is simply applied to one bias factor and does not involve principal component regression. Moreover, in contrast to their usage of the log enrichment of read counts in multiple lanes as response variables, our method is able to correct for the absolute gene expression levels even when there is no technical replicates on multiple lanes.

It is well known that due to the random sampling nature of RNA-Seq data, length normalized gene level expressions exhibit larger sample variance for shorter genes [9]. Since our method considered each sample separately, we have not solved the unequal variance problem, which is important for detecting differentially expressed genes between samples. Moreover, the length bias we detected here is different from the bias detected by Oshlack and Wakefield [9]. We observed a general trend of lower median RPKM/FPKM for longer genes, whereas Oshlack and Wakefield reported lower variance of gene level read counts for longer genes. Under the assumption of random sampling, the gene expression level in RPKM/FPKM unit (essentially the gene level read counts normalized by gene length) should be unbiased in terms of gene length, therefore our observation indicates potential deviation from random sampling model.

## Conclusions

In summary, our work identified several potential biases in RNA-Seq data, and proposed a bias correction method that can provide a more accurate gene level expression estimate. We believe this method will help meta-analysis of RNA-Seq data with other quantitative platforms and alternative protocols.

## Competing interests

The authors declare that they have no competing interests.

## Authors' contributions

WZ performed statistical analysis, wrote the RNASeqBias package and drafted the manuscript. LC participated in the data analysis and R package development. HZ conceived of the study, designed and coordinated the study and helped to draft the manuscript. All authors read and approved the final manuscript.

## Supplementary Material

Additional file 1 **Summary of data processing procedures for each data set**. Click here for file

Additional file 2 **R package RNASeqBias developed in this study**. Click here for file

Additional file 3 **Bias plots for Marioni, Mamanova, Lee and Nagalakshmi data sets (Procedure 1, gene-level)**. Click here for file

Additional file 4 **Bias plots for MAQC data using transcript level expression summaries**. Click here for file

Additional file 5 **Bias plots for Marioni data using Procedures 2 and 3**. Click here for file

Additional file 6 **Bias plots for SINO genes in MAQC data**. Click here for file

Additional file 7 **P-values of testing whether bias patterns are platform-specific or sample-specific**. Click here for file

Additional file 8 **PCA plots for 8 data sets**. The left panel shows the variance explained by each principal component. Black bars are PCs included in the GAM model (they explain at least 95% variance in the data), and gray bars are PCs not included. The right panel shows the biplot for the first two PCs. The length of each red vector represents the standard deviation of each predictor, and the angle between any two vectors represents the correlation between them. The relative orientation to x and y axis shows the relationship between individual predictors and the first two PCs. The inset table shows the non-parametric F-statistic for each predictor in the fitted GAM model and the corresponding p-value. Click here for file

Additional file 9 **Improvement of cross-platform correlations with different number of PCs in GAM**. GAM models using gene length only or length plus 1-7 PCs were fitted to correct the MAQC2 brain and UHR RNA-Seq data set, and the correlations (r) between uncorrected/corrected seq data and RT-PCR/QuantiGene data were calculated. Click here for file

Additional file 10 **Scatter plots with different GAM terms**. Correlations between corrected log(RPKM) using (length + GC content) and corrected log(RPKM) using 1, 2, 3, or minimal number of PCs explaining 95% of the variance. Subfigures A-H are plots for 8 data sets in the same order as in Additional file 5. Click here for file

Additional file 11 **Plots of the component smooth functions that make up the fitted GAM objects with two predictors: gene length and GC content**. The left panel shows the 1-D smooth for log gene length, and the right panel shows the 1-D smooth for GC content. The rug plot at the bottom of each panel shows the values of each predictor. The dotted lines are 2-standard errors above and below the estimated smooth (solid lines). Subfigures A-H are plots for 8 data sets in the same order as in Additional file 5. Click here for file

Additional file 12 **Bias plots for MAQC data using Procedure 3 and comparing with random hexamer bias correction method**. Click here for file

Additional file 13 **Bias plots for SINO genes in Lee data using Procedure 3 and comparing with random hexamer bias correction method and mseq method**. Click here for file

Additional file 14 **Comparison of correlations between RT-PCR and MAQC2 RNA-Seq data**. Gene expression levels from RT-PCR are compared with original RNA-Seq data (Procedure 3), RNA-Seq data with random hexamer bias correction, and RNA-Seq data with GAM correction. Click here for file
