# Supplementary material for: Bias detection and correction in RNA-Sequencing data
Source: BMC Bioinformatics. 2011 Jul 19;12:290. doi: 10.1186/1471-2105-12-290 (PMC3149584; doi:10.1186/1471-2105-12-290)
Supplement: Additional file 2 — R package RNASeqBias developed in this study. [file 1471-2105-12-290-S2.GZ › RNASeqBias/inst/doc/vignette.pdf]

Bias Detection and Correction in RNA-Sequencing Data

Wei Zheng, Lisa Chung, Hongyu Zhao

March 7, 2011

1 Install the package and load a test data set

To load the RNASeqBias package, type

```
> library(RNASeqBias)
```

To get the sample data set, type

```
> data(MAQCtestdata)
> allCounts <- data.frame(Counts, reweightedCounts)
```

The MAQCtestdata is a list with four components, counts mapped to each ENSEMBL transcript in one MAQC2 experiment (Counts), counts after prime reweighting adjustment (reweightedCounts), values of bias factors for each transcript (tanno) and information of single-isoform non-overlapping genes (sinog) based on hg19 ENS59 annotation. The brain data is from SRR037452, and the UHR data is from SRR037459. The counts data and corresponding RPKM values before and after prime reweighting can also be generated from alignment files using the function prime.reweight.counts in the package.

To convert counts to transcript level expression in RPKM unit, we need to multiply the counts by 10^9, and divide by total number of counts and gene length.

```
> trExp <- apply(allCounts * 10^9, 1, `/`, colSums(Counts))
> tl <- tanno[match(rownames(Counts), tanno[, 2]), 3]
> trExp <- apply(trExp, 1, `/`, tl)
> colnames(trExp) <- c("BRAIN", "UHR", "BRAIN.RW", "UHR.RW")
```

For gene-level data, we used median transcript level expression as gene level expression.

```
> gene <- as.character(tanno[match(rownames(trExp), tanno[, 2]),
+     1])
> geneExp <- apply(trExp, 2, function(X) {
+     ave(X, gene, FUN = median)
+ })
> geneExp <- data.frame(gene, geneExp)
> geneExp <- unique(geneExp)
> colnames(geneExp)[-1] <- colnames(trExp)
```

For gene-level data, we used median transcript level bias factors as gene level bias factors.

```
> ganno <- apply(tanno[, 3:20], 2, function(X) {
+     ave(X, tanno[, 1], FUN = median)
+ })
> ganno <- data.frame(tanno[, 1], ganno)
> ganno <- unique(ganno)
> rownames(ganno) <- ganno[, 1]
> ganno <- ganno[, -1]
```

For single isoform non-overlapping genes,

```
> sinoExp <- geneExp[match(sinog[, 1], geneExp[, 1]), ]
> dim(trExp)

[1] 142467      4

> dim(geneExp)

[1] 49733     5

> dim(sinoExp)

[1] 20555     5
```

To filter out genes not present in any samples,

```
> trExp <- trExp[apply(trExp, 1, sum) > 0, ]
> geneExp <- geneExp[apply(geneExp[, -1], 1, sum) > 0, ]
> sinoExp <- sinoExp[apply(sinoExp[, -1], 1, sum) > 0, ]
> dim(trExp)

[1] 119699      4

> dim(geneExp)

[1] 32484     5

> dim(sinoExp)

[1] 9314    5
```

2 Checking bias plots and correcting for biases using **gampc** function

For transcript level,

```
> biasanno <- tanno[, -(1:2)]
> rownames(biasanno) <- tanno[, 2]
> Exp <- trExp
> grpnn <- 500
```

For gene level,

```
> biasanno <- ganno
> Exp <- geneExp
> rownames(Exp) <- geneExp[, 1]
> Exp <- Exp[, -1]
> grpnn <- 200
```

For single isoform non-overlapping gene level,

```
> biasanno <- ganno[as.character(sinog[, 1]), ]
> Exp <- sinoExp
> rownames(Exp) <- sinoExp[, 1]
> Exp <- Exp[, -1]
> grpnn <- 40
```

With any of the above calculated biasanno, Exp and grpnn, we can plot and correct for the biases using the following code, and store the corrected values in the list gamcrt.

```
> bias <- biasanno[rownames(Exp), ]
> bias[, 1] <- log(bias[, 1])
> colnames(bias)[1] <- "Log_L"
> trend_bias <- gamcrt <- list()
> for (i in 1:4) {
+     zeroid <- which(Exp[, i] == 0)
+     trend_bias[[i]] <- group_plot(cbind(log(Exp[-zeroid, i]),
+         bias[-zeroid, ]), grpn = grpnn)
+     gamcrt[[i]] <- gampc(log(Exp[-zeroid, i]), bias[-zeroid,
+         1], bias[-zeroid, 2:18])
+     trend_bias[[i + 4]] <- group_plot(cbind(gamcrt[[i]], bias[-zeroid,
+         ]), grpn = grpnn)
+ }
> group_plotm(trend_bias, minval = -2, maxval = 1.5, titl = colnames(bias))
> legend("bottomright", c("Brain", "UHR", "Brain_RW", "UHR_RW",
+     "Brain GAM", "UHR GAM", "Brain_RW GAM", "UHR_RW GAM"), col = 1:8,
+     lty = 1, cex = 1.2)
```

3 References

1. Hansen KD, Brenner SE, Dudoit S: Biases in Illumina transcriptome sequencing caused by random hexamer priming. *Nucleic Acids Res* 2010, **38**(12):e131.
