# Supplementary material for: Bias detection and correction in RNA-Sequencing data
Source: BMC Bioinformatics. 2011 Jul 19;12:290. doi: 10.1186/1471-2105-12-290 (PMC3149584; doi:10.1186/1471-2105-12-290)
Supplement: Additional file 3 — Bias plots for Marioni, Mamanova, Lee and Nagalakshmi data sets (Procedure 1, gene-level). [file 1471-2105-12-290-S3.PPT]

## Slide 1
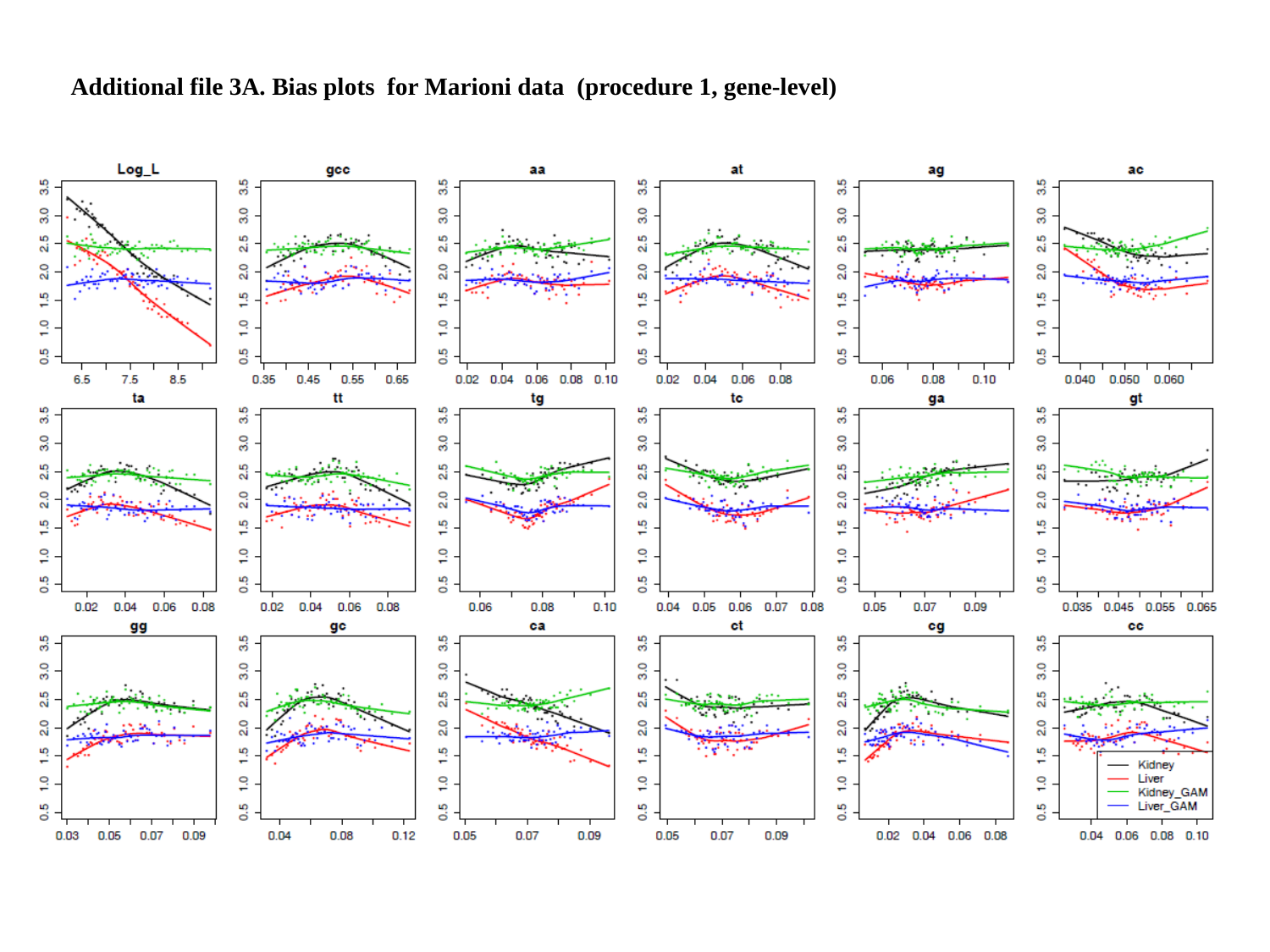

Additional file 3A. Bias plots for Marioni data (procedure 1, gene-level)

## Slide 2
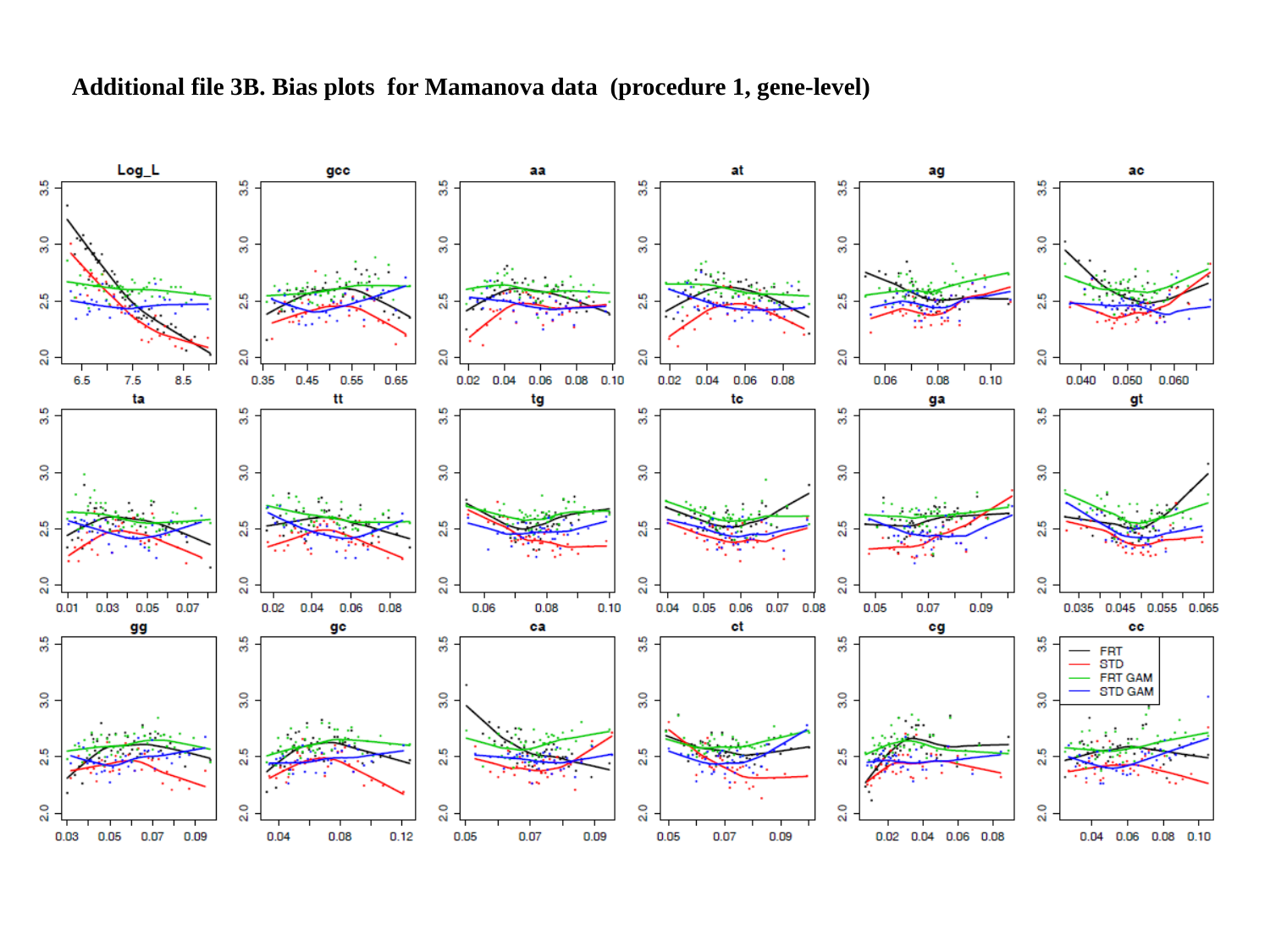

Additional file 3B. Bias plots for Mamanova data (procedure 1, gene-level)

## Slide 3
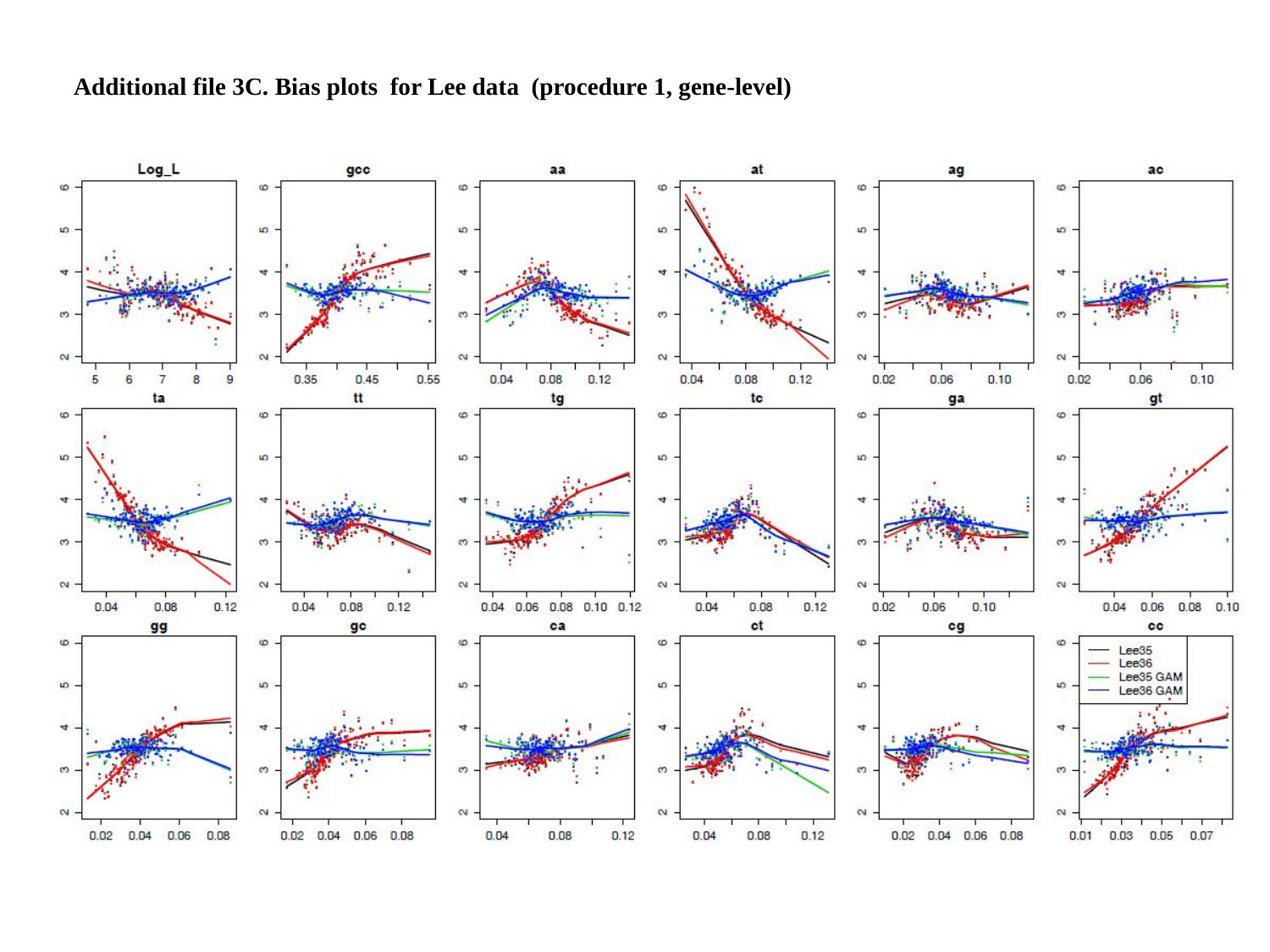

Additional file 3C. Bias plots for Lee data (procedure 1, gene-level)

## Slide 4
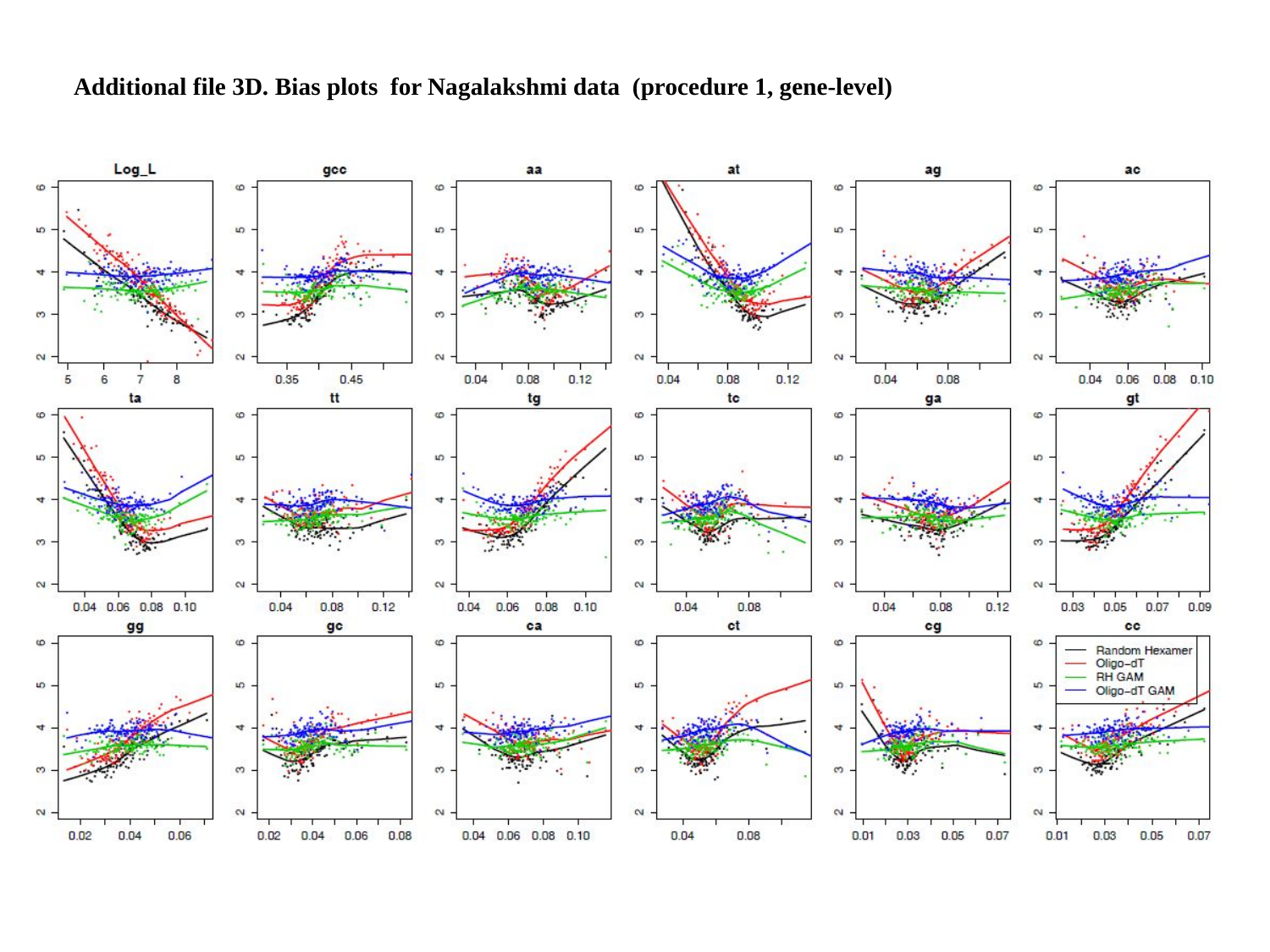

Additional file 3D. Bias plots for Nagalakshmi data (procedure 1, gene-level)
