# Supplementary material for: Bias detection and correction in RNA-Sequencing data
Source: BMC Bioinformatics. 2011 Jul 19;12:290. doi: 10.1186/1471-2105-12-290 (PMC3149584; doi:10.1186/1471-2105-12-290)
Supplement: Additional file 4 — Bias plots for MAQC data using transcript level expression summaries. [file 1471-2105-12-290-S4.PPT]

## Slide 1
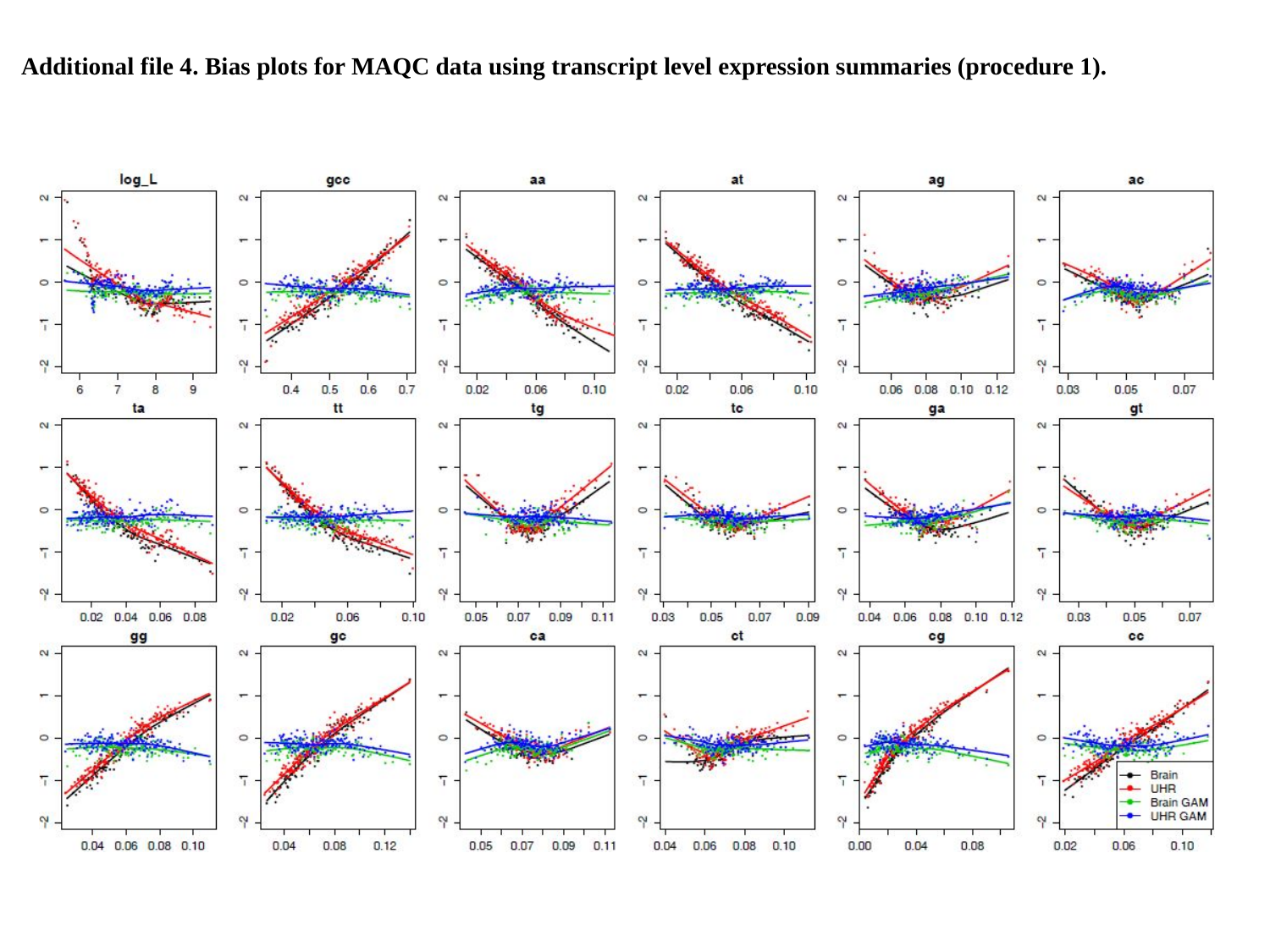

Additional file 4. Bias plots for MAQC data using transcript level expression summaries (procedure 1).
