# Supplementary material for: Bias detection and correction in RNA-Sequencing data
Source: BMC Bioinformatics. 2011 Jul 19;12:290. doi: 10.1186/1471-2105-12-290 (PMC3149584; doi:10.1186/1471-2105-12-290)
Supplement: Additional file 5 — Bias plots for Marioni data using Procedures 2 and 3. [file 1471-2105-12-290-S5.PPT]

## Slide 1
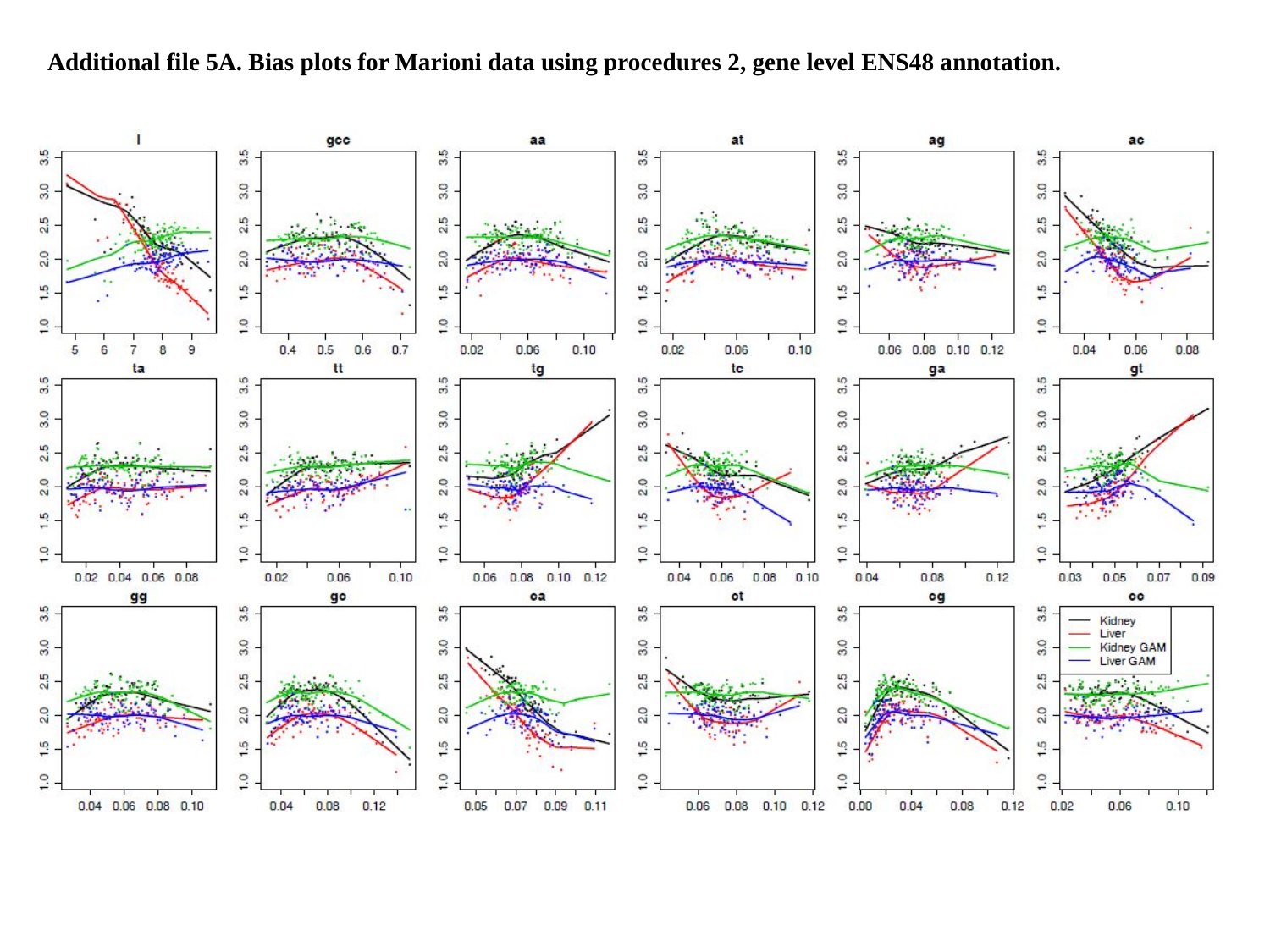

Additional file 5A. Bias plots for Marioni data using procedures 2, gene level ENS48 annotation.

## Slide 2
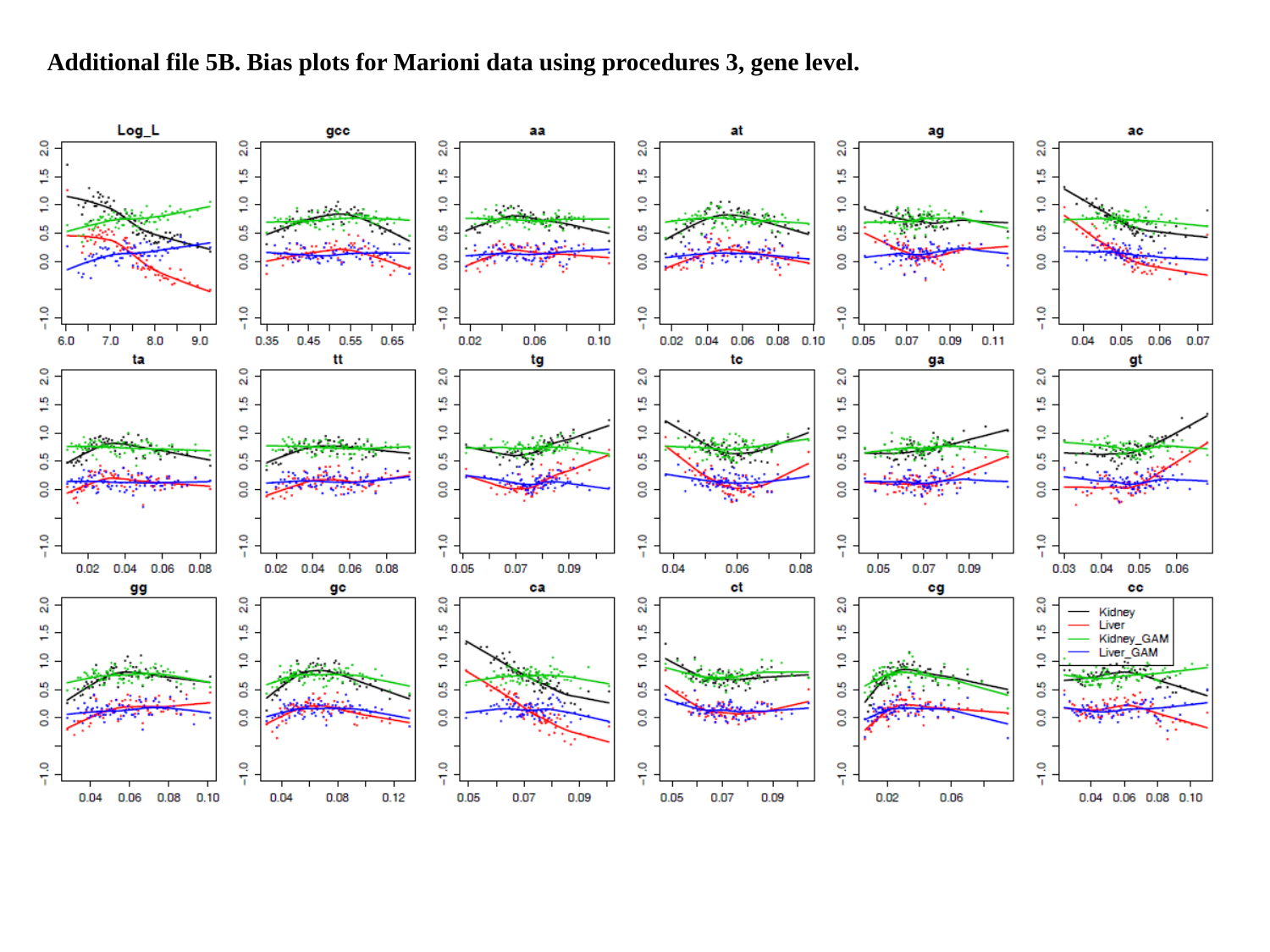

Additional file 5B. Bias plots for Marioni data using procedures 3, gene level.
