# Supplementary figures and images for: Bias detection and correction in RNA-Sequencing data
Source: BMC Bioinformatics. 2011 Jul 19;12:290. doi: 10.1186/1471-2105-12-290 (PMC3149584; doi:10.1186/1471-2105-12-290)

## Slide 1
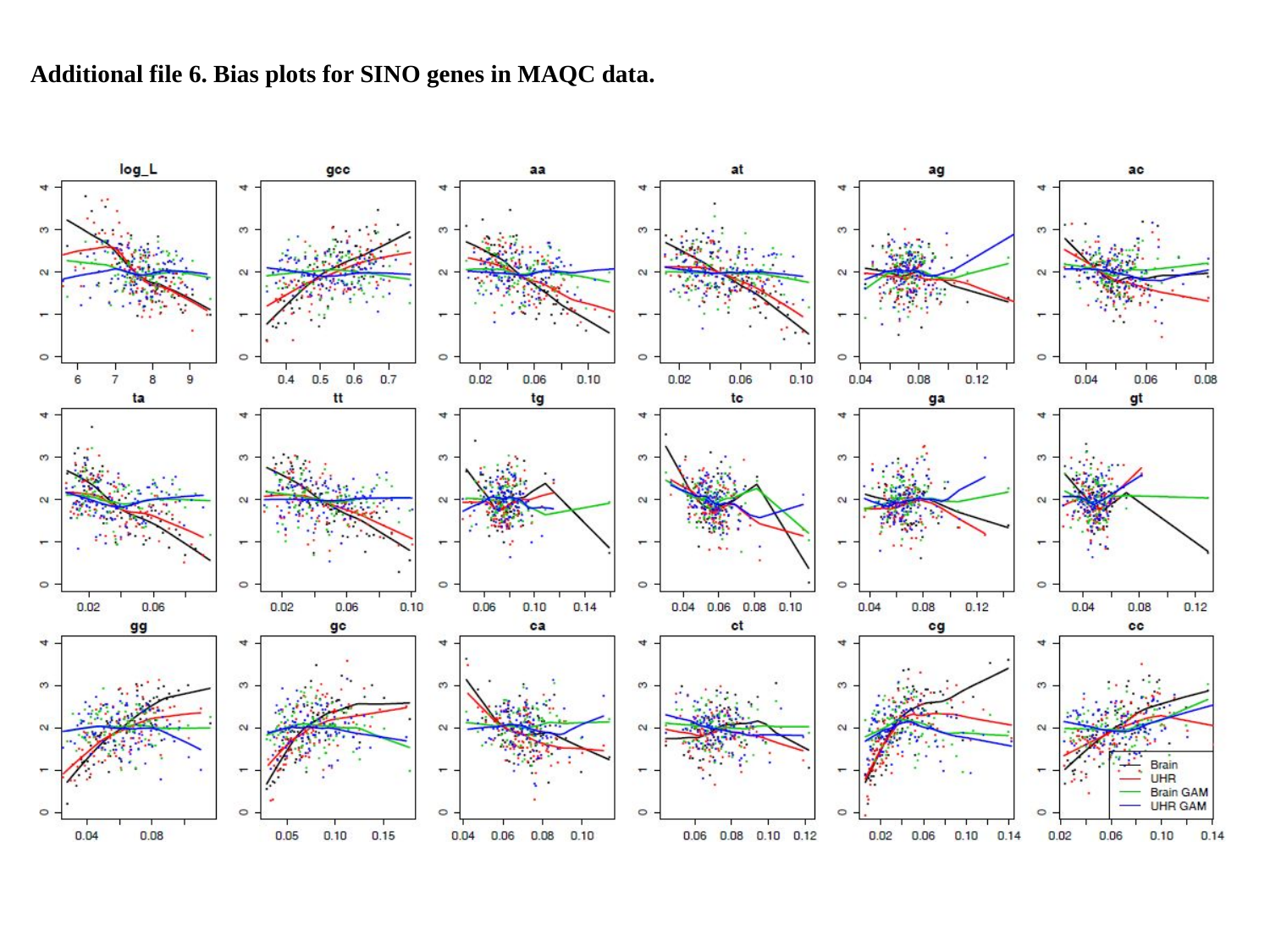

Additional file 6. Bias plots for SINO genes in MAQC data.

Supplement: Additional file 6 — Bias plots for SINO genes in MAQC data. [file 1471-2105-12-290-S6.PPT]
