# Supplementary material for: Bias detection and correction in RNA-Sequencing data
Source: BMC Bioinformatics. 2011 Jul 19;12:290. doi: 10.1186/1471-2105-12-290 (PMC3149584; doi:10.1186/1471-2105-12-290)
Supplement: Additional file 8 — PCA plots for 8 data sets. The left panel shows the variance explained by each principal component. Black bars are PCs included in the GAM model (they explain at least 95% variance in the data), and gray bars are PCs not included. The right panel shows the biplot for the first two PCs. The length of each red vector represents the standard deviation of each predictor, and the angle between any two vectors represents the correlation between them. The relative orientation to x and y axis shows the relationship between individual predictors and the first two PCs. The inset table shows the non-parametric F-statistic for each predictor in the fitted GAM model and the corresponding p-value. [file 1471-2105-12-290-S8.PPT]

## Slide 1
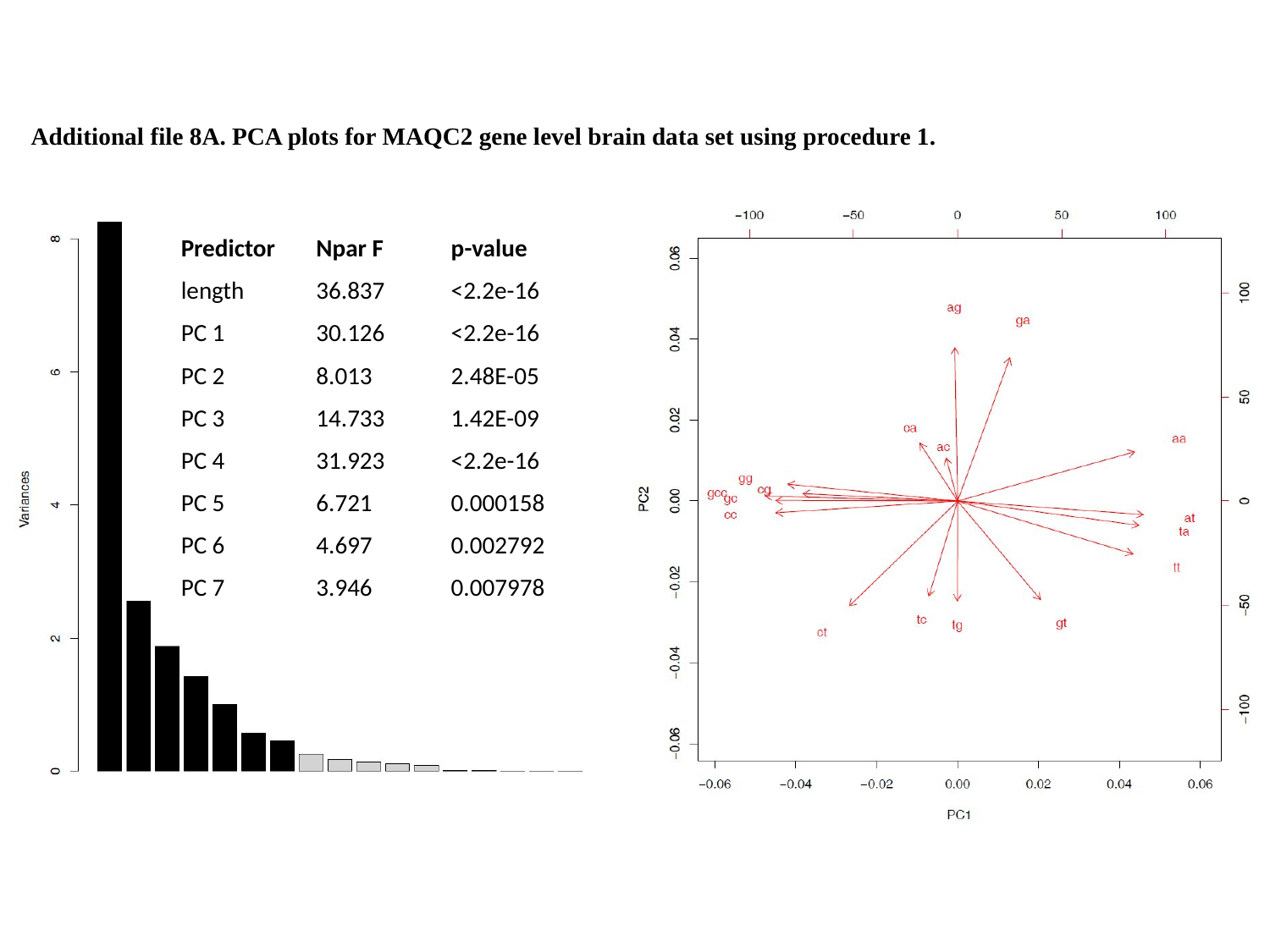

Additional file 8A. PCA plots for MAQC2 gene level brain data set using procedure 1.
| Predictor | Npar F | p-value |
| --- | --- | --- |
| length | 36.837 | <2.2e-16 |
| PC 1 | 30.126 | <2.2e-16 |
| PC 2 | 8.013 | 2.48E-05 |
| PC 3 | 14.733 | 1.42E-09 |
| PC 4 | 31.923 | <2.2e-16 |
| PC 5 | 6.721 | 0.000158 |
| PC 6 | 4.697 | 0.002792 |
| PC 7 | 3.946 | 0.007978 |

## Slide 2
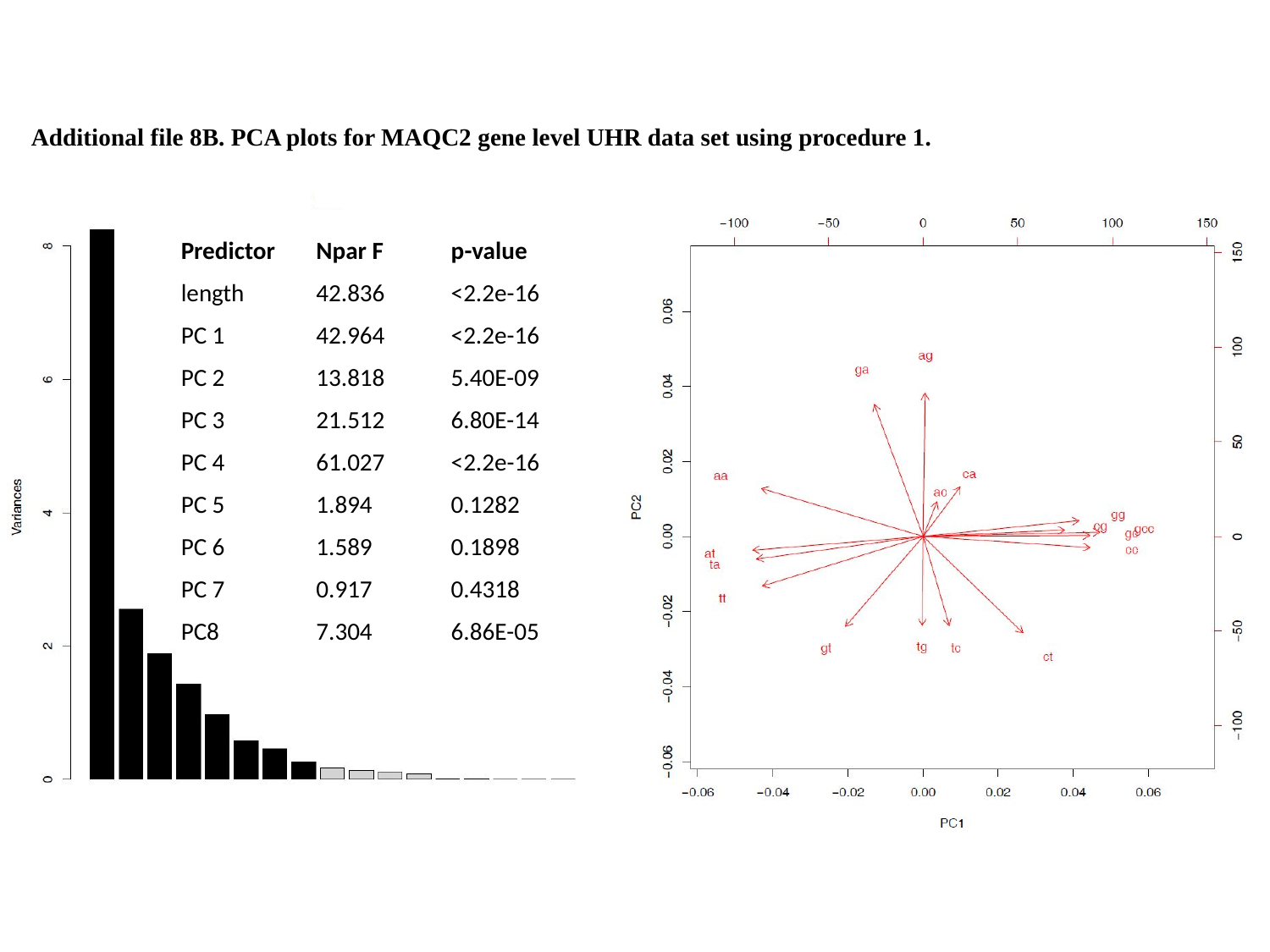

Additional file 8B. PCA plots for MAQC2 gene level UHR data set using procedure 1.
| Predictor | Npar F | p-value |
| --- | --- | --- |
| length | 42.836 | <2.2e-16 |
| PC 1 | 42.964 | <2.2e-16 |
| PC 2 | 13.818 | 5.40E-09 |
| PC 3 | 21.512 | 6.80E-14 |
| PC 4 | 61.027 | <2.2e-16 |
| PC 5 | 1.894 | 0.1282 |
| PC 6 | 1.589 | 0.1898 |
| PC 7 | 0.917 | 0.4318 |
| PC8 | 7.304 | 6.86E-05 |

## Slide 3
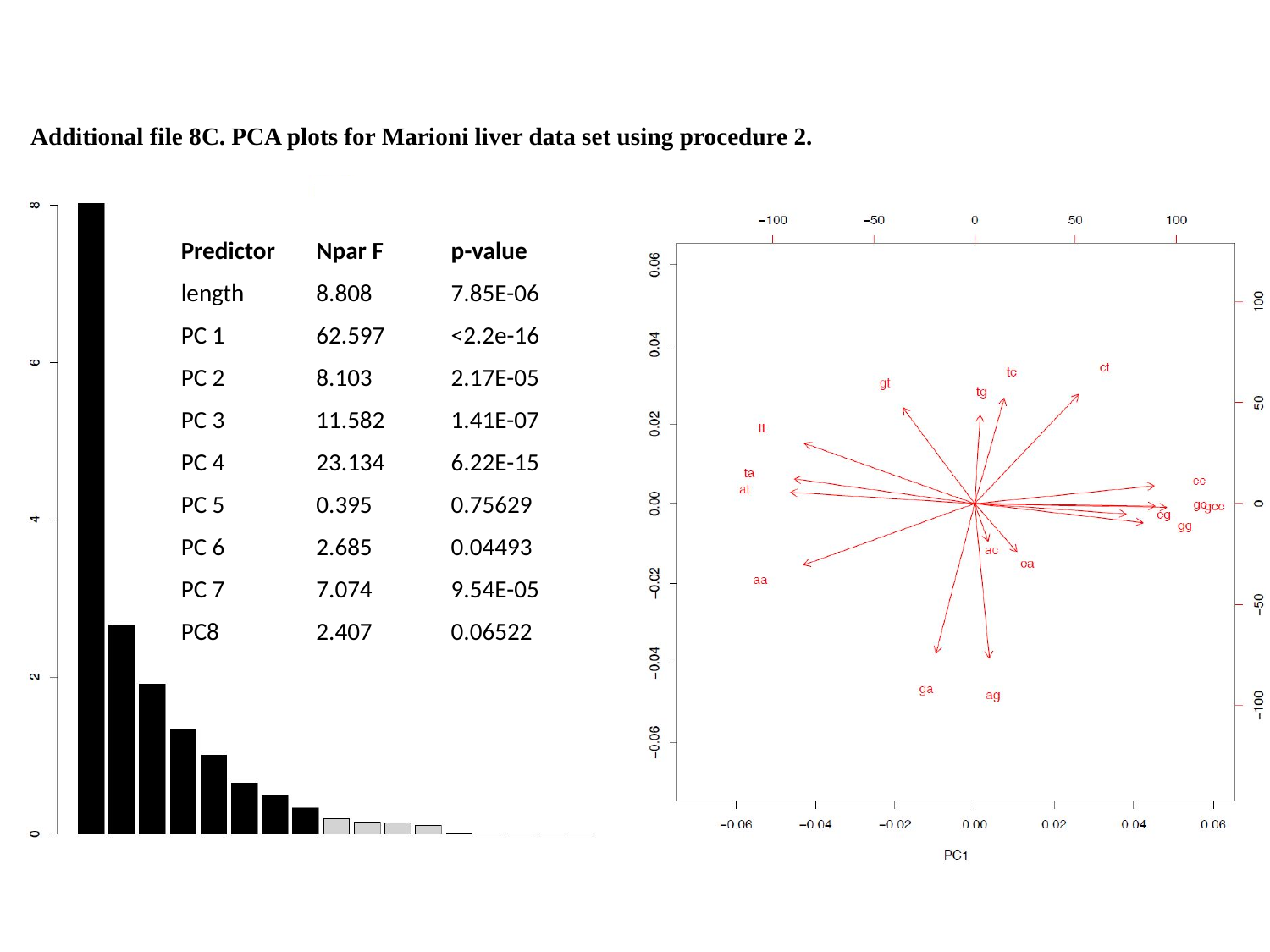

Additional file 8C. PCA plots for Marioni liver data set using procedure 2.
| Predictor | Npar F | p-value |
| --- | --- | --- |
| length | 8.808 | 7.85E-06 |
| PC 1 | 62.597 | <2.2e-16 |
| PC 2 | 8.103 | 2.17E-05 |
| PC 3 | 11.582 | 1.41E-07 |
| PC 4 | 23.134 | 6.22E-15 |
| PC 5 | 0.395 | 0.75629 |
| PC 6 | 2.685 | 0.04493 |
| PC 7 | 7.074 | 9.54E-05 |
| PC8 | 2.407 | 0.06522 |

## Slide 4
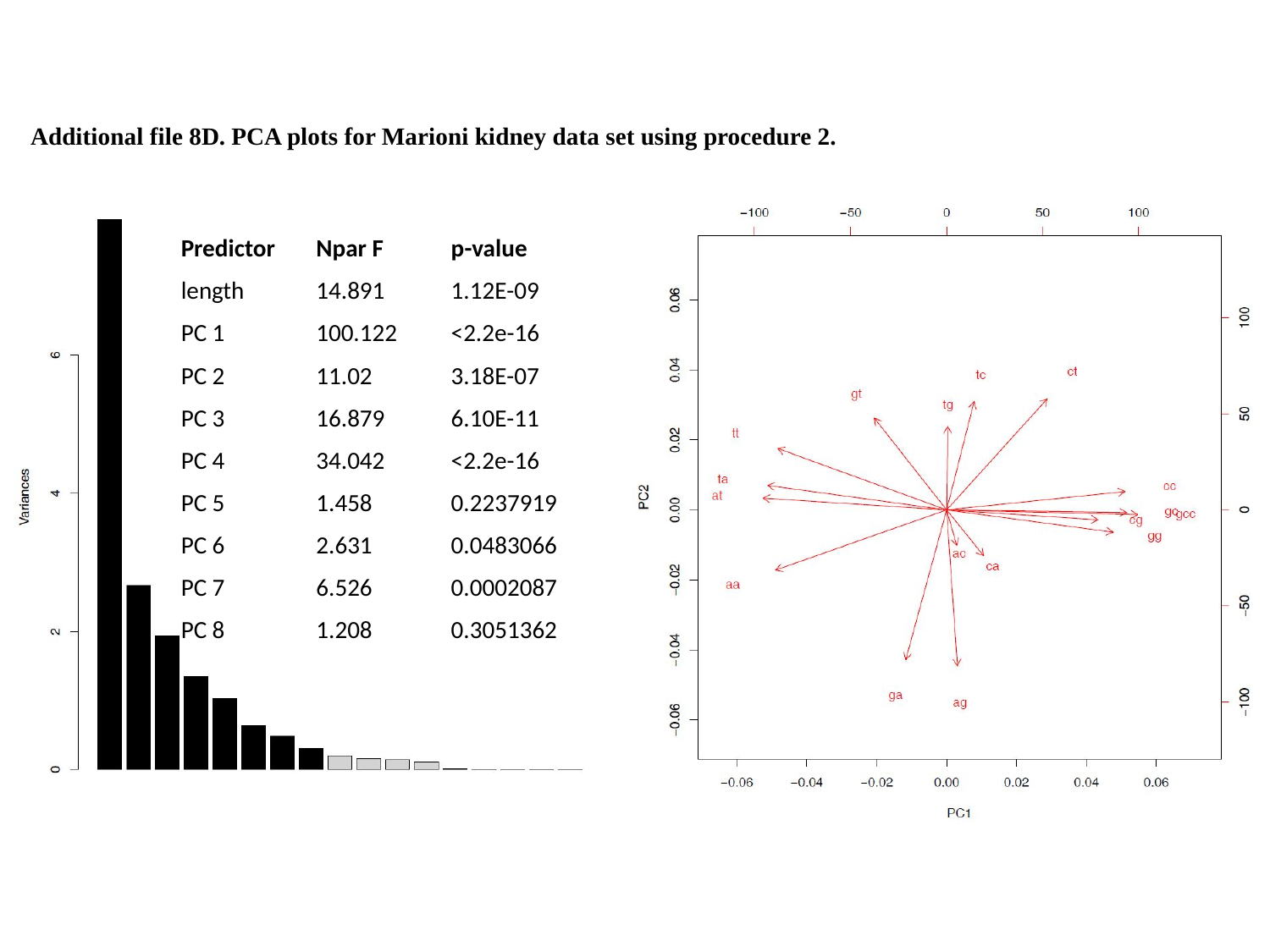

Additional file 8D. PCA plots for Marioni kidney data set using procedure 2.
| Predictor | Npar F | p-value |
| --- | --- | --- |
| length | 14.891 | 1.12E-09 |
| PC 1 | 100.122 | <2.2e-16 |
| PC 2 | 11.02 | 3.18E-07 |
| PC 3 | 16.879 | 6.10E-11 |
| PC 4 | 34.042 | <2.2e-16 |
| PC 5 | 1.458 | 0.2237919 |
| PC 6 | 2.631 | 0.0483066 |
| PC 7 | 6.526 | 0.0002087 |
| PC 8 | 1.208 | 0.3051362 |

## Slide 5
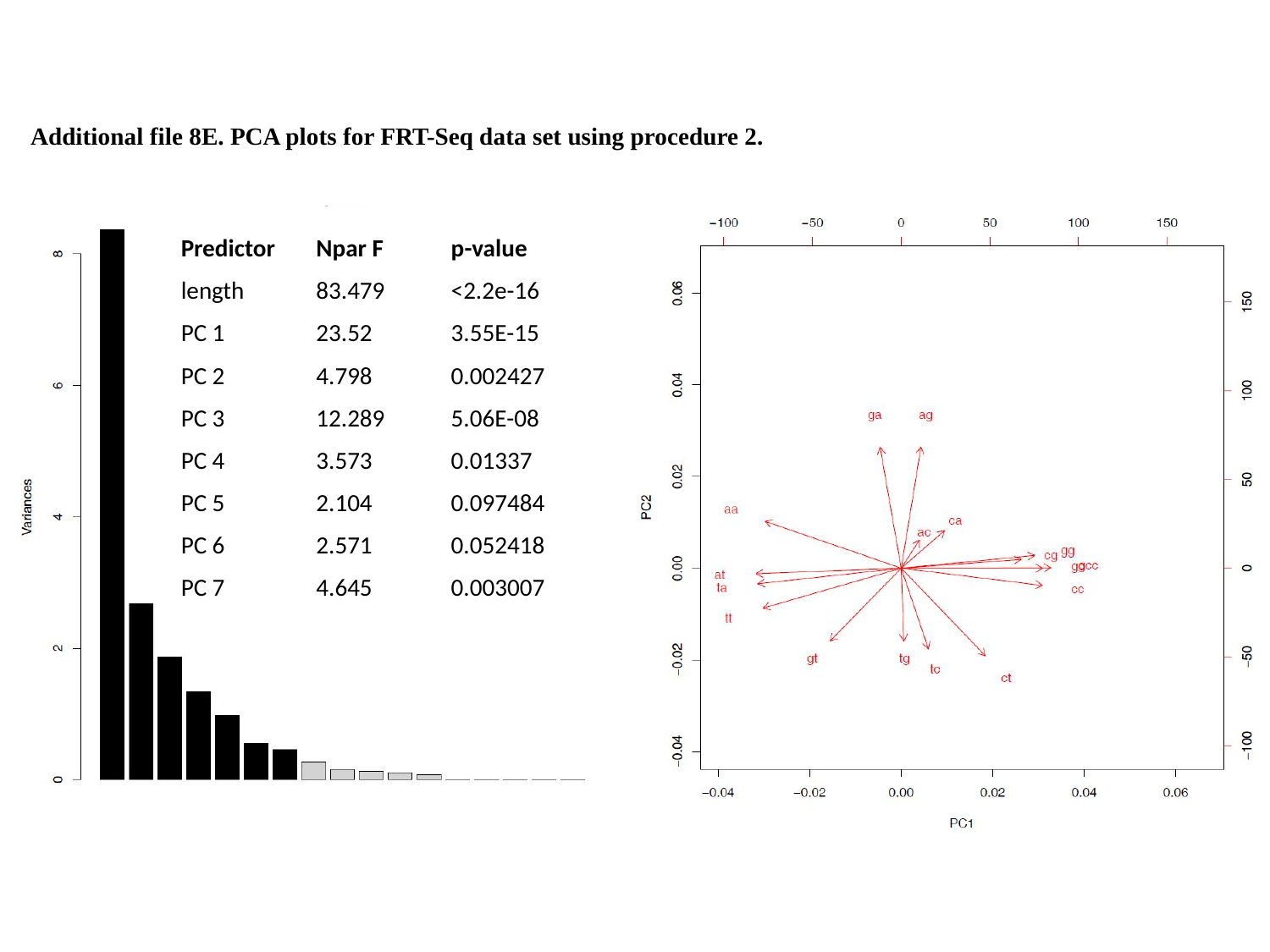

Additional file 8E. PCA plots for FRT-Seq data set using procedure 2.
| Predictor | Npar F | p-value |
| --- | --- | --- |
| length | 83.479 | <2.2e-16 |
| PC 1 | 23.52 | 3.55E-15 |
| PC 2 | 4.798 | 0.002427 |
| PC 3 | 12.289 | 5.06E-08 |
| PC 4 | 3.573 | 0.01337 |
| PC 5 | 2.104 | 0.097484 |
| PC 6 | 2.571 | 0.052418 |
| PC 7 | 4.645 | 0.003007 |

## Slide 6
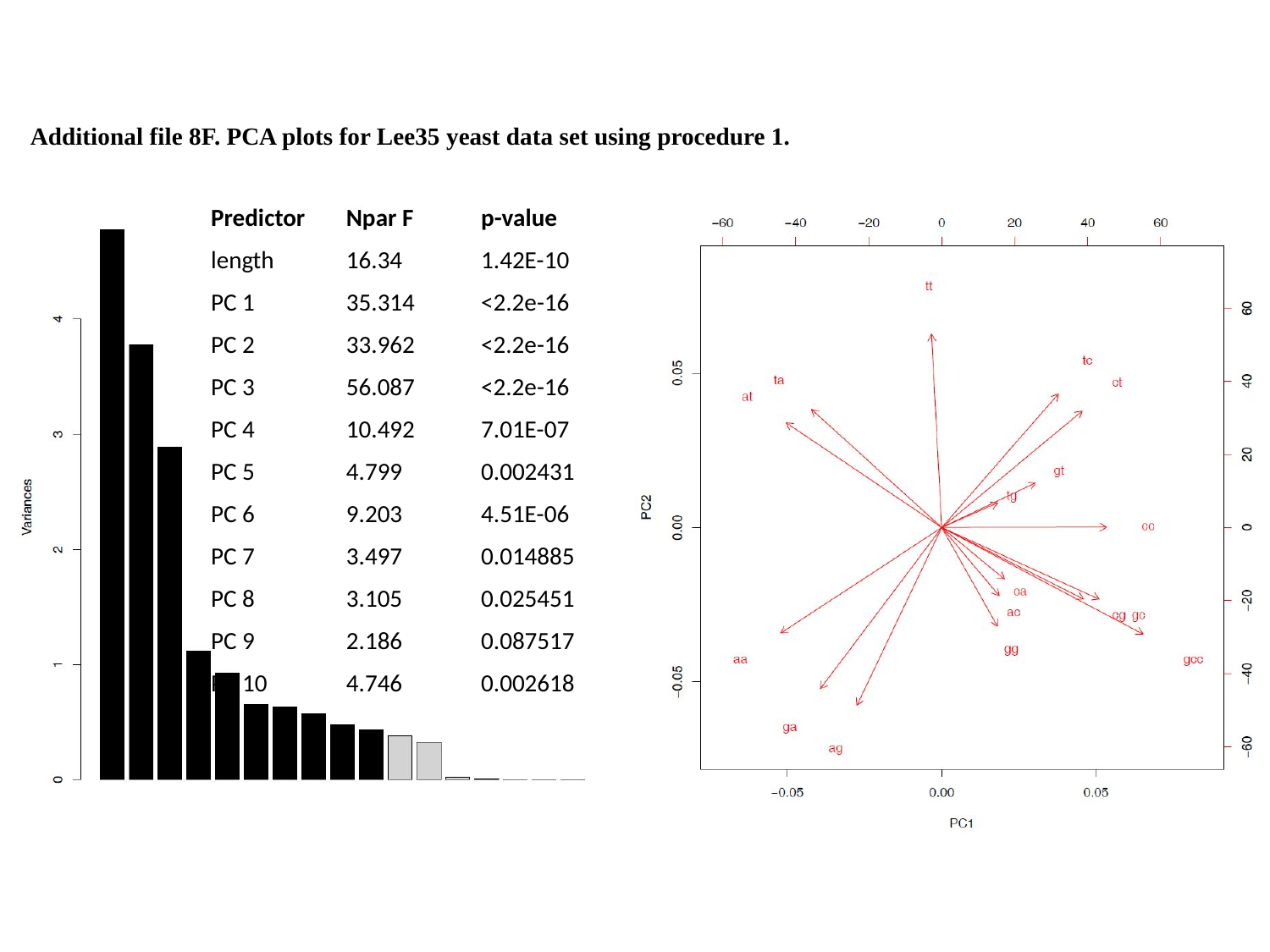

Additional file 8F. PCA plots for Lee35 yeast data set using procedure 1.
| Predictor | Npar F | p-value |
| --- | --- | --- |
| length | 16.34 | 1.42E-10 |
| PC 1 | 35.314 | <2.2e-16 |
| PC 2 | 33.962 | <2.2e-16 |
| PC 3 | 56.087 | <2.2e-16 |
| PC 4 | 10.492 | 7.01E-07 |
| PC 5 | 4.799 | 0.002431 |
| PC 6 | 9.203 | 4.51E-06 |
| PC 7 | 3.497 | 0.014885 |
| PC 8 | 3.105 | 0.025451 |
| PC 9 | 2.186 | 0.087517 |
| PC 10 | 4.746 | 0.002618 |

## Slide 7
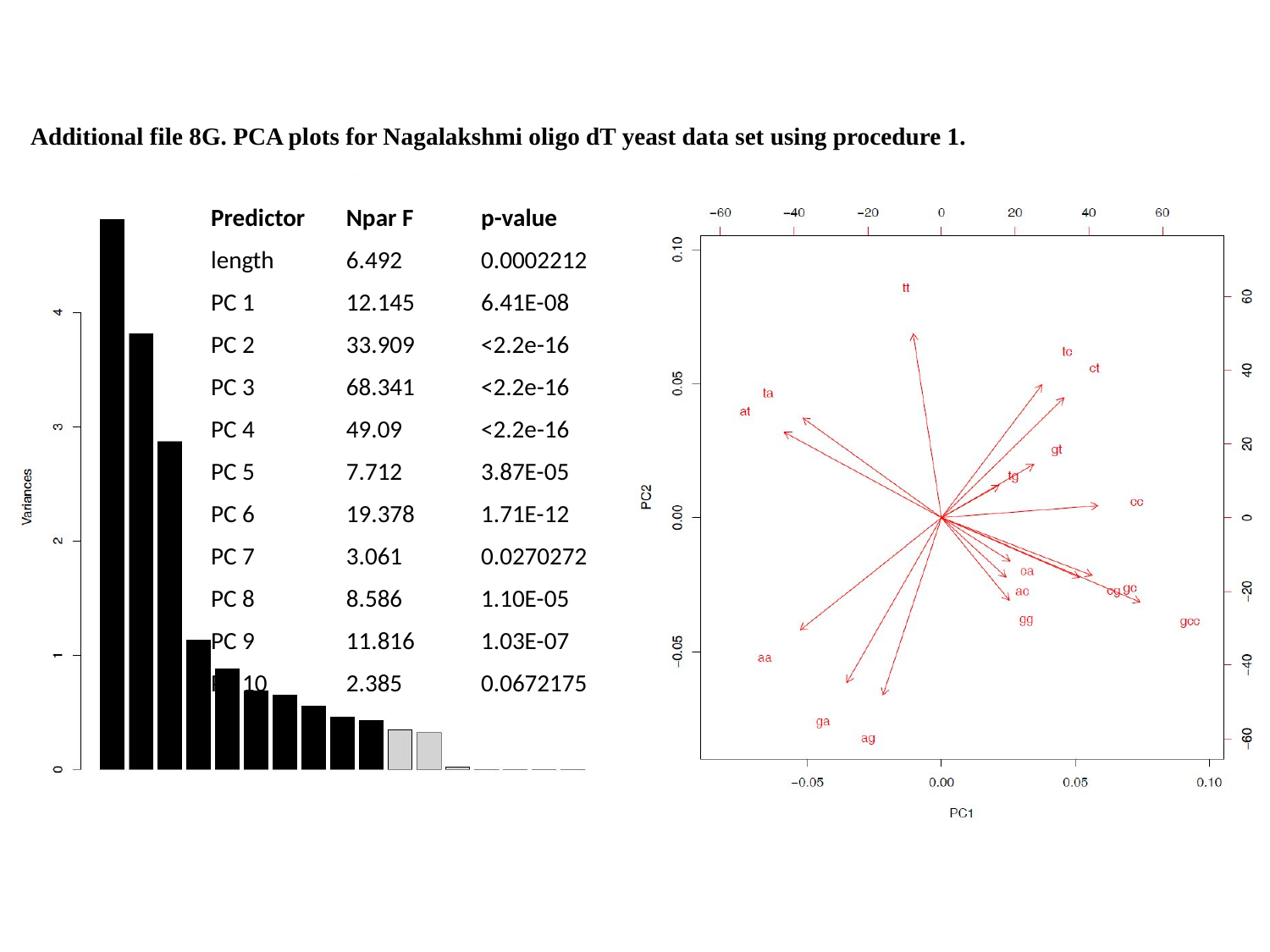

Additional file 8G. PCA plots for Nagalakshmi oligo dT yeast data set using procedure 1.
| Predictor | Npar F | p-value |
| --- | --- | --- |
| length | 6.492 | 0.0002212 |
| PC 1 | 12.145 | 6.41E-08 |
| PC 2 | 33.909 | <2.2e-16 |
| PC 3 | 68.341 | <2.2e-16 |
| PC 4 | 49.09 | <2.2e-16 |
| PC 5 | 7.712 | 3.87E-05 |
| PC 6 | 19.378 | 1.71E-12 |
| PC 7 | 3.061 | 0.0270272 |
| PC 8 | 8.586 | 1.10E-05 |
| PC 9 | 11.816 | 1.03E-07 |
| PC 10 | 2.385 | 0.0672175 |

## Slide 8
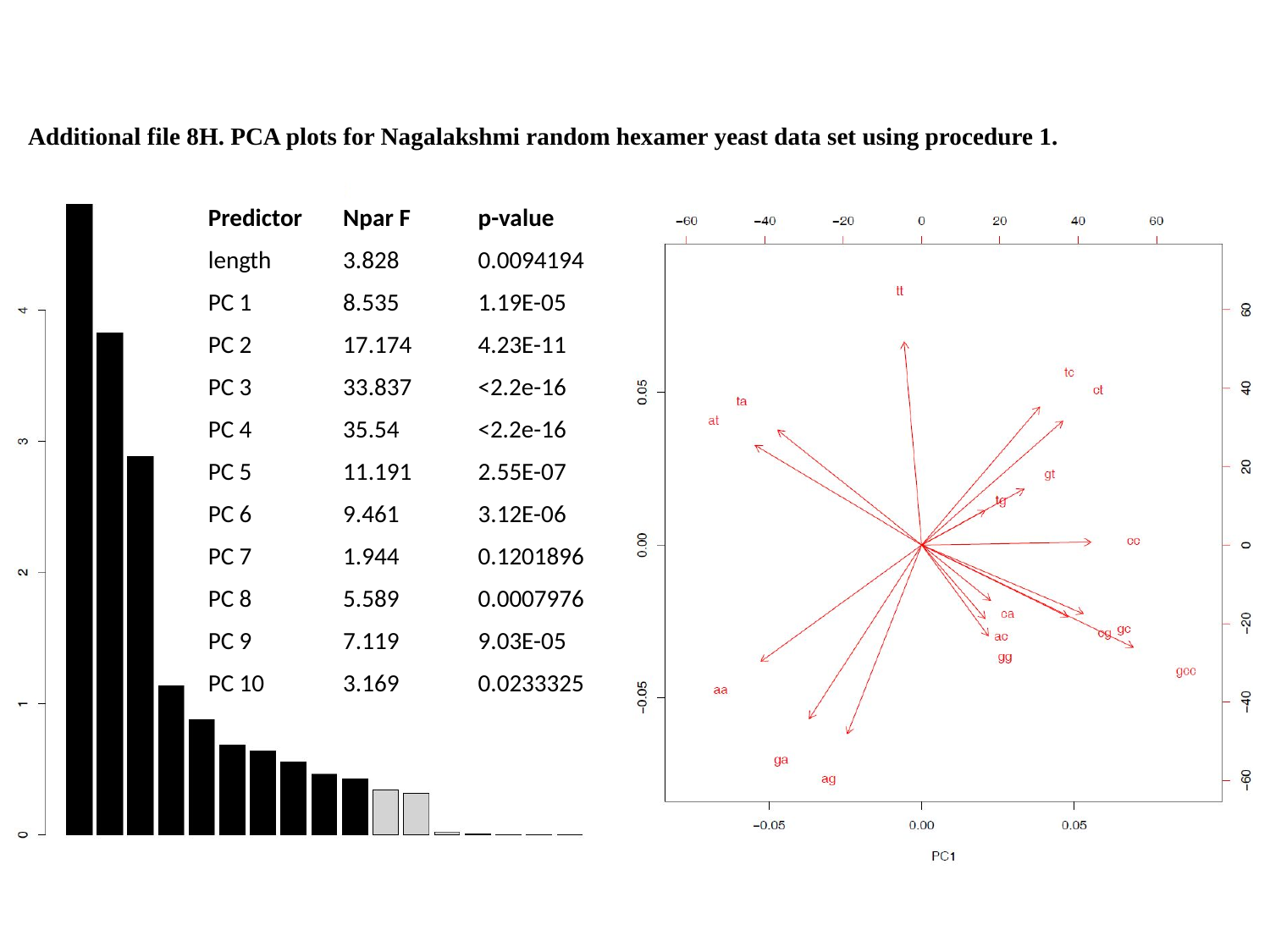

Additional file 8H. PCA plots for Nagalakshmi random hexamer yeast data set using procedure 1.
| Predictor | Npar F | p-value |
| --- | --- | --- |
| length | 3.828 | 0.0094194 |
| PC 1 | 8.535 | 1.19E-05 |
| PC 2 | 17.174 | 4.23E-11 |
| PC 3 | 33.837 | <2.2e-16 |
| PC 4 | 35.54 | <2.2e-16 |
| PC 5 | 11.191 | 2.55E-07 |
| PC 6 | 9.461 | 3.12E-06 |
| PC 7 | 1.944 | 0.1201896 |
| PC 8 | 5.589 | 0.0007976 |
| PC 9 | 7.119 | 9.03E-05 |
| PC 10 | 3.169 | 0.0233325 |
