# Supplementary material for: Bias detection and correction in RNA-Sequencing data
Source: BMC Bioinformatics. 2011 Jul 19;12:290. doi: 10.1186/1471-2105-12-290 (PMC3149584; doi:10.1186/1471-2105-12-290)
Supplement: Additional file 9 — Improvement of cross-platform correlations with different number of PCs in GAM. GAM models using gene length only or length plus 1-7 PCs were fitted to correct the MAQC2 brain and UHR RNA-Seq data set, and the correlations (r) between uncorrected/corrected seq data and RT-PCR/QuantiGene data were calculated. [file 1471-2105-12-290-S9.PPT]

## Slide 1
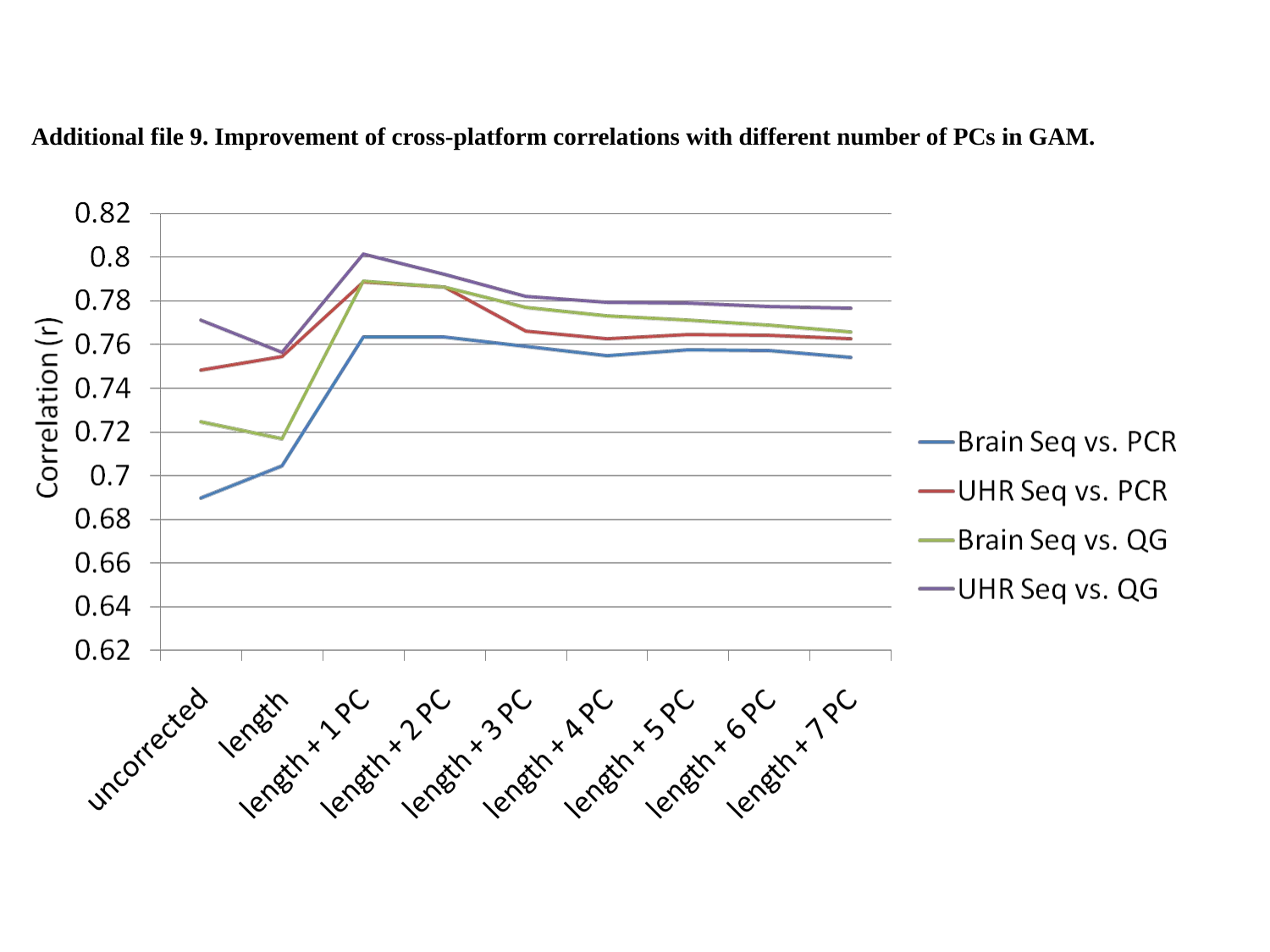

Additional file 9. Improvement of cross-platform correlations with different number of PCs in GAM.
