# Supplementary material for: Bias detection and correction in RNA-Sequencing data
Source: BMC Bioinformatics. 2011 Jul 19;12:290. doi: 10.1186/1471-2105-12-290 (PMC3149584; doi:10.1186/1471-2105-12-290)
Supplement: Additional file 10 — Scatter plots with different GAM terms. Correlations between corrected log(RPKM) using (length + GC content) and corrected log(RPKM) using 1, 2, 3, or minimal number of PCs explaining 95% of the variance. Subfigures A-H are plots for 8 data sets in the same order as in Additional file 5. [file 1471-2105-12-290-S10.PPT]

## Slide 1
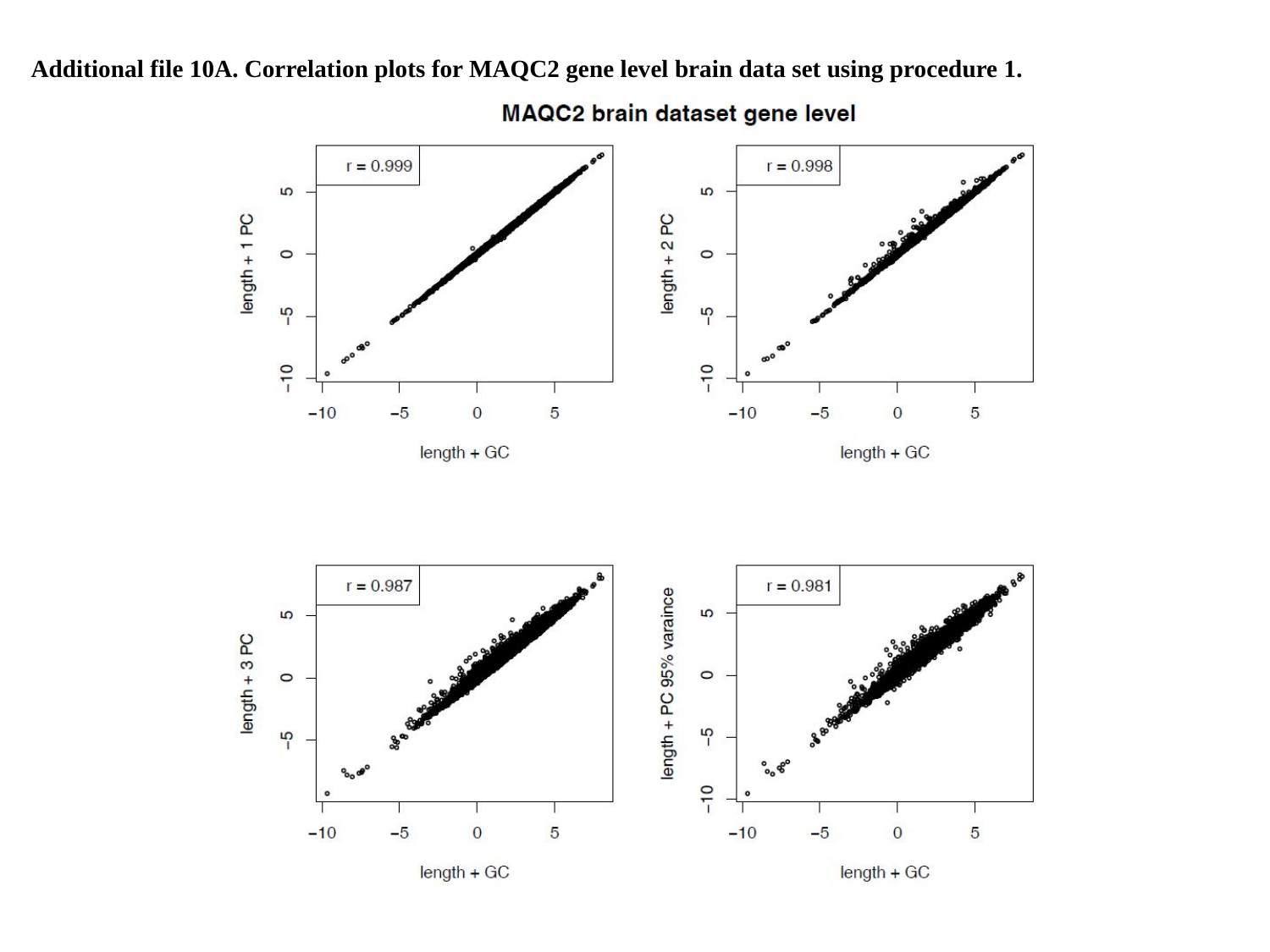

Additional file 10A. Correlation plots for MAQC2 gene level brain data set using procedure 1.

## Slide 2
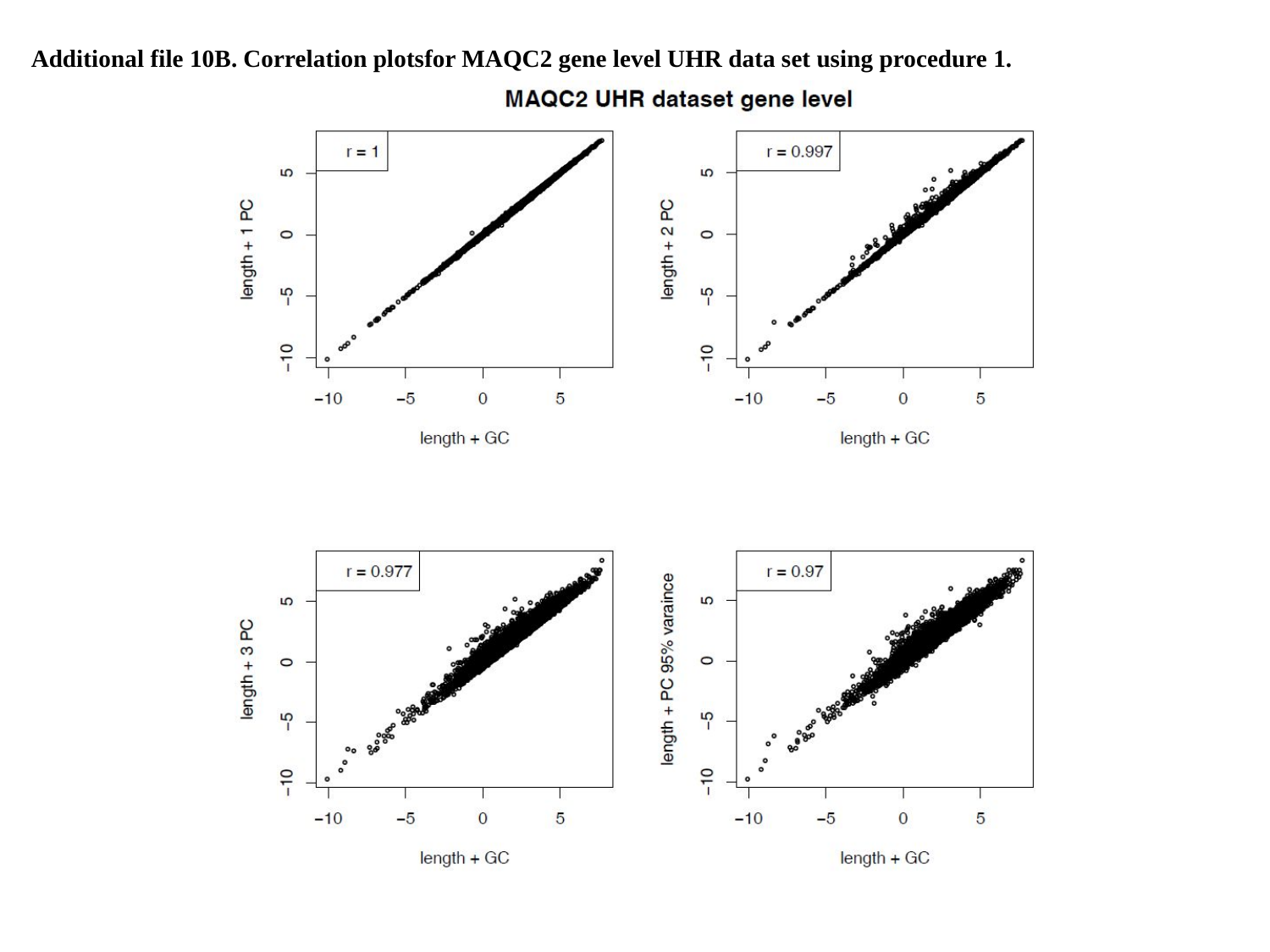

Additional file 10B. Correlation plotsfor MAQC2 gene level UHR data set using procedure 1.

## Slide 3
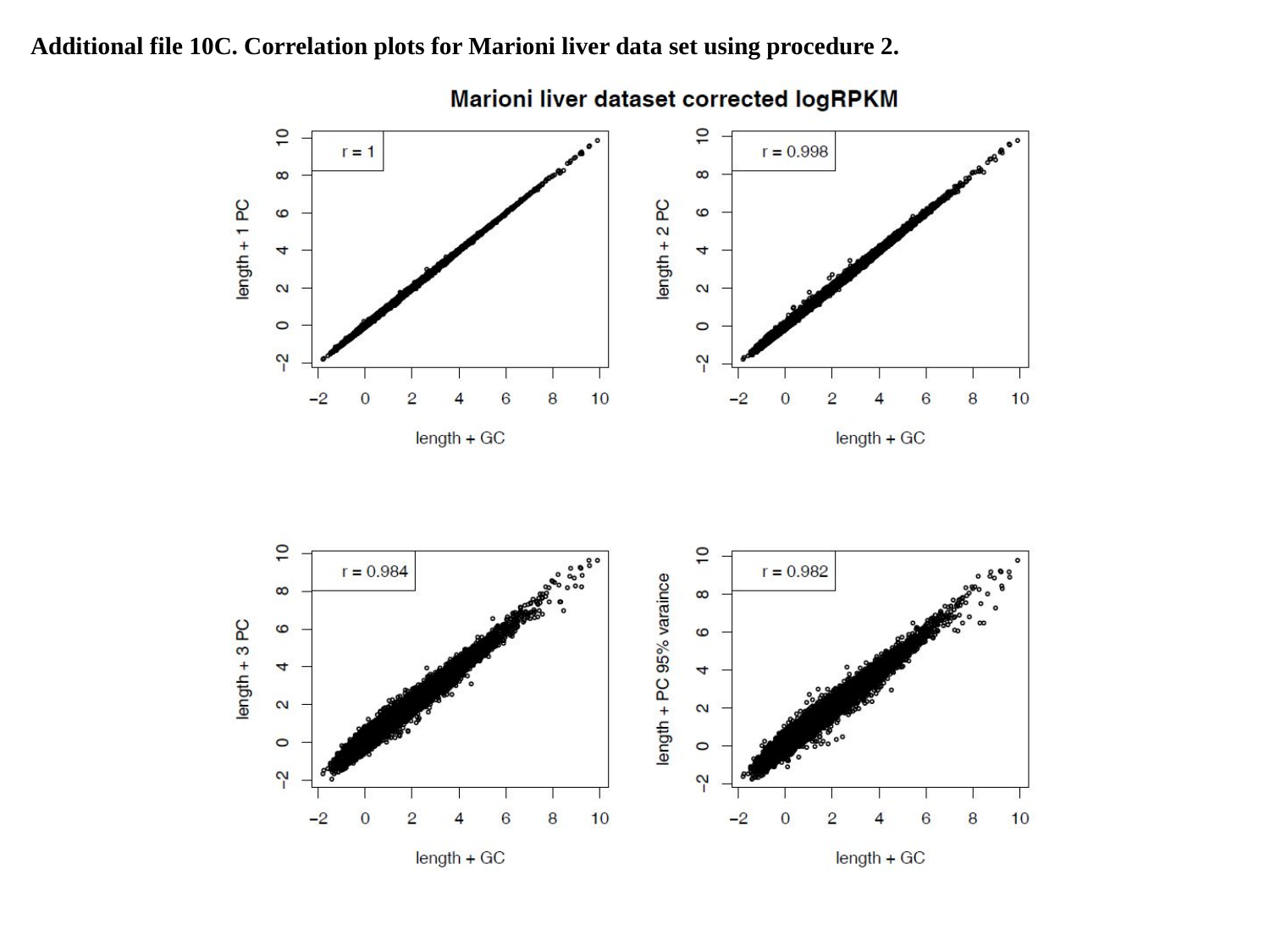

Additional file 10C. Correlation plots for Marioni liver data set using procedure 2.

## Slide 4
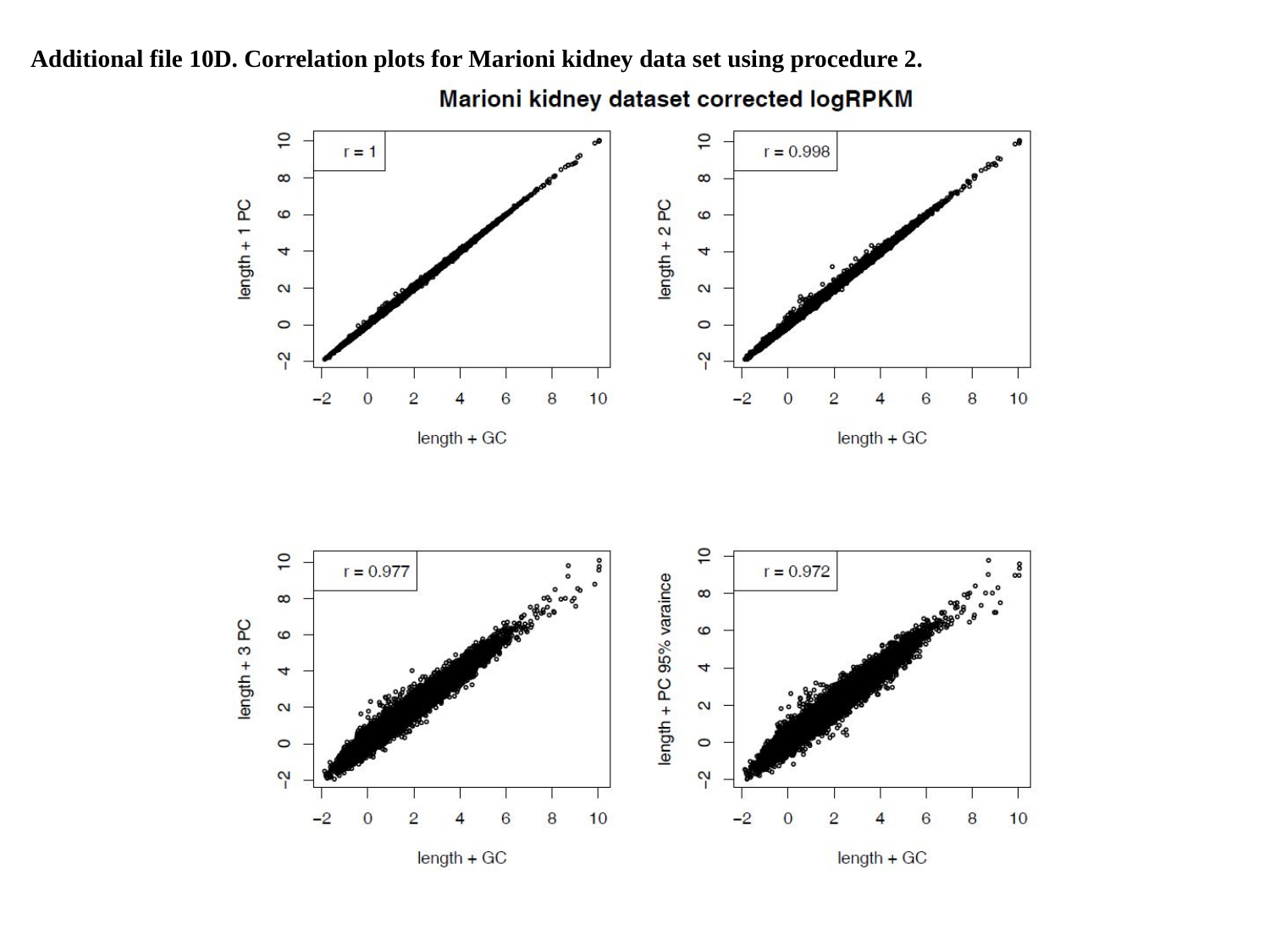

Additional file 10D. Correlation plots for Marioni kidney data set using procedure 2.

## Slide 5
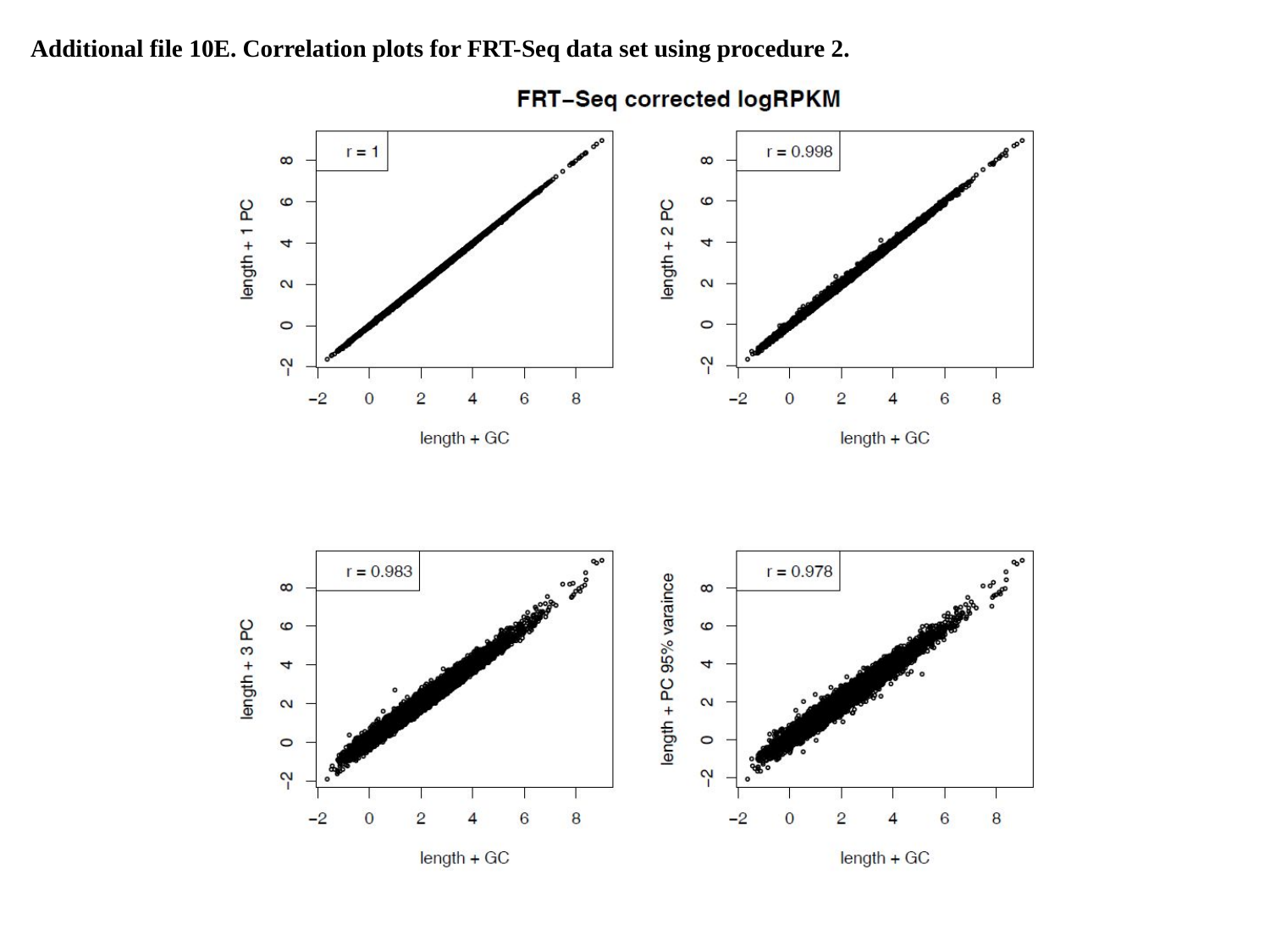

Additional file 10E. Correlation plots for FRT-Seq data set using procedure 2.

## Slide 6
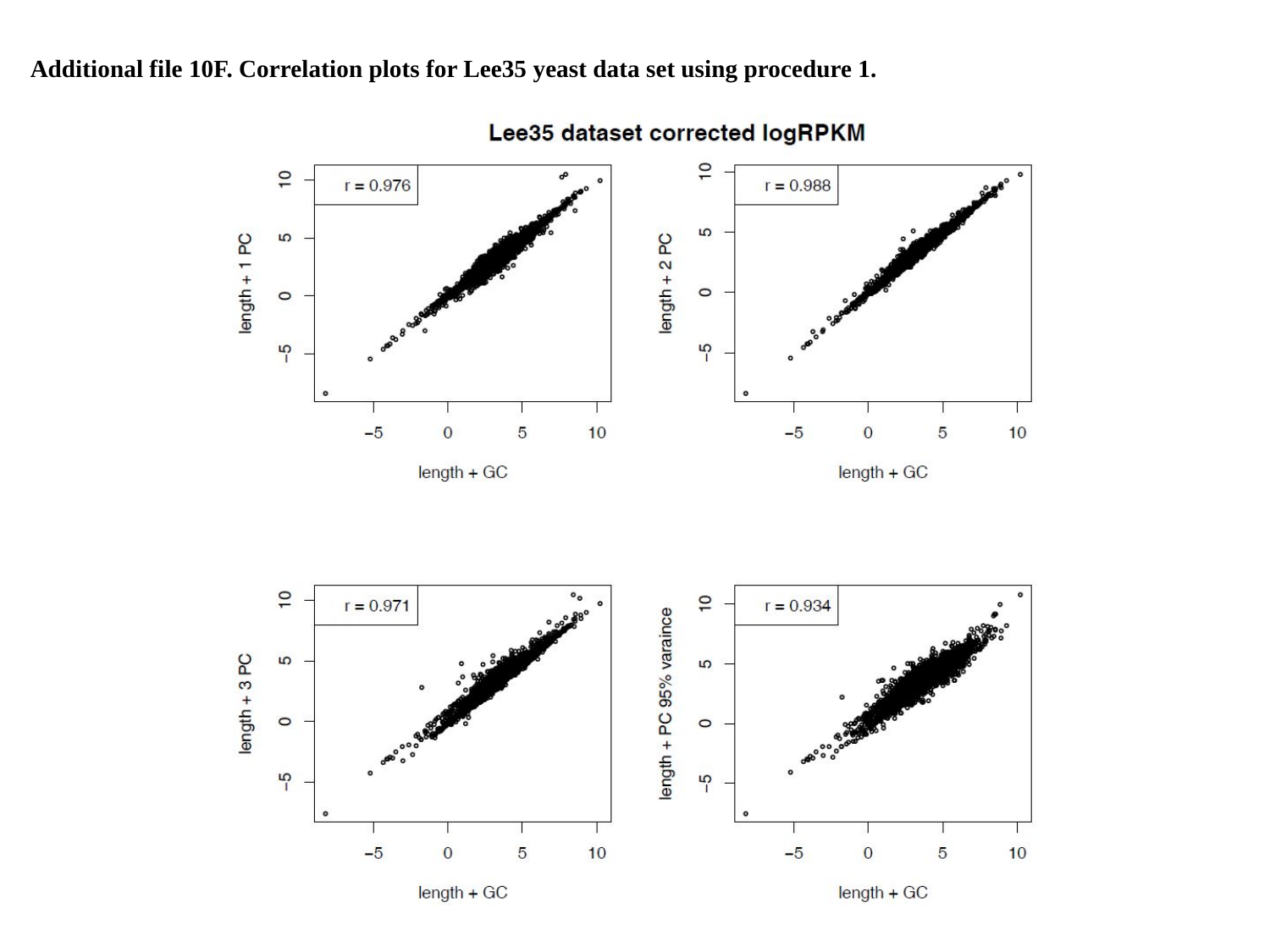

Additional file 10F. Correlation plots for Lee35 yeast data set using procedure 1.

## Slide 7
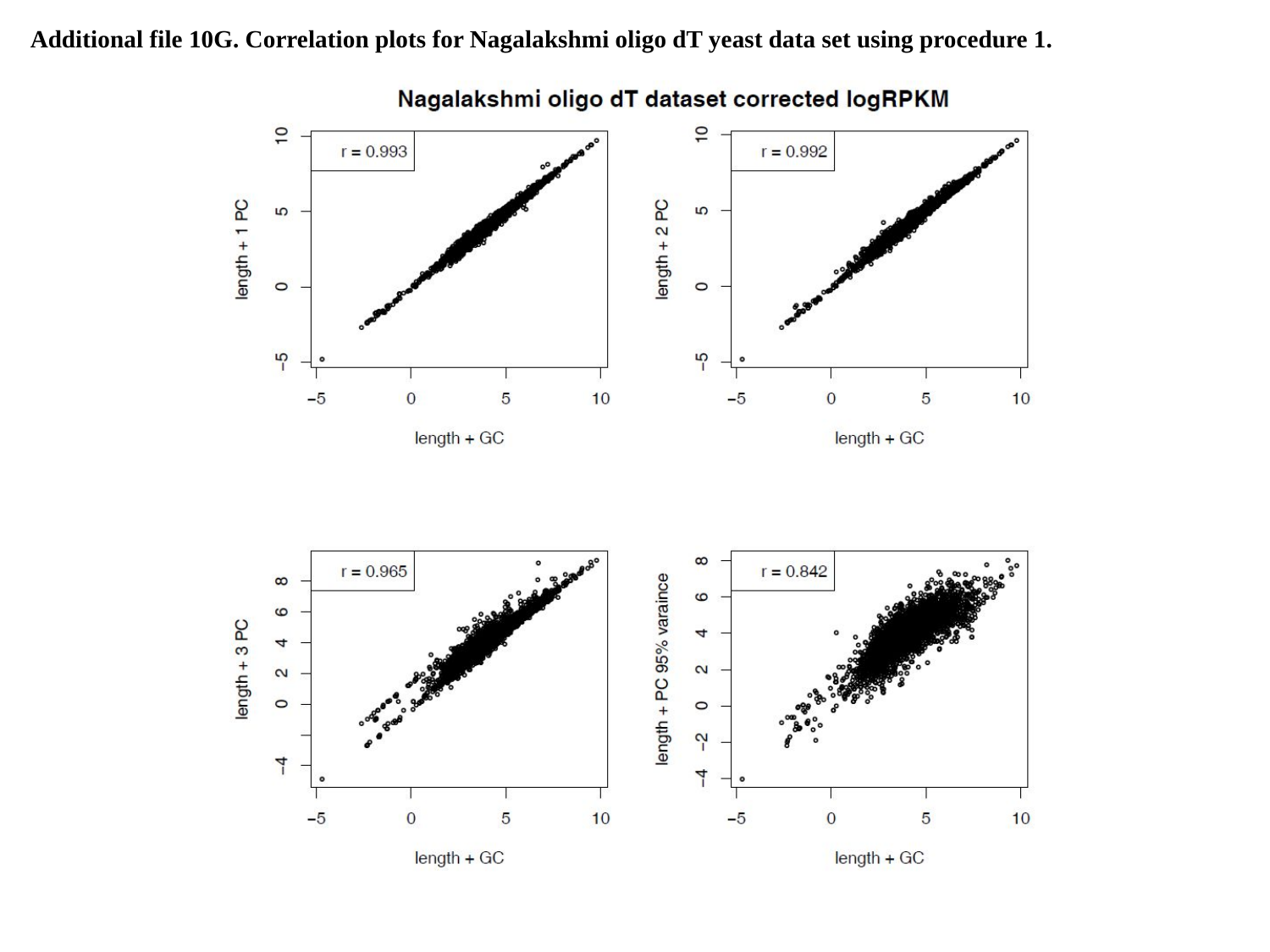

Additional file 10G. Correlation plots for Nagalakshmi oligo dT yeast data set using procedure 1.

## Slide 8
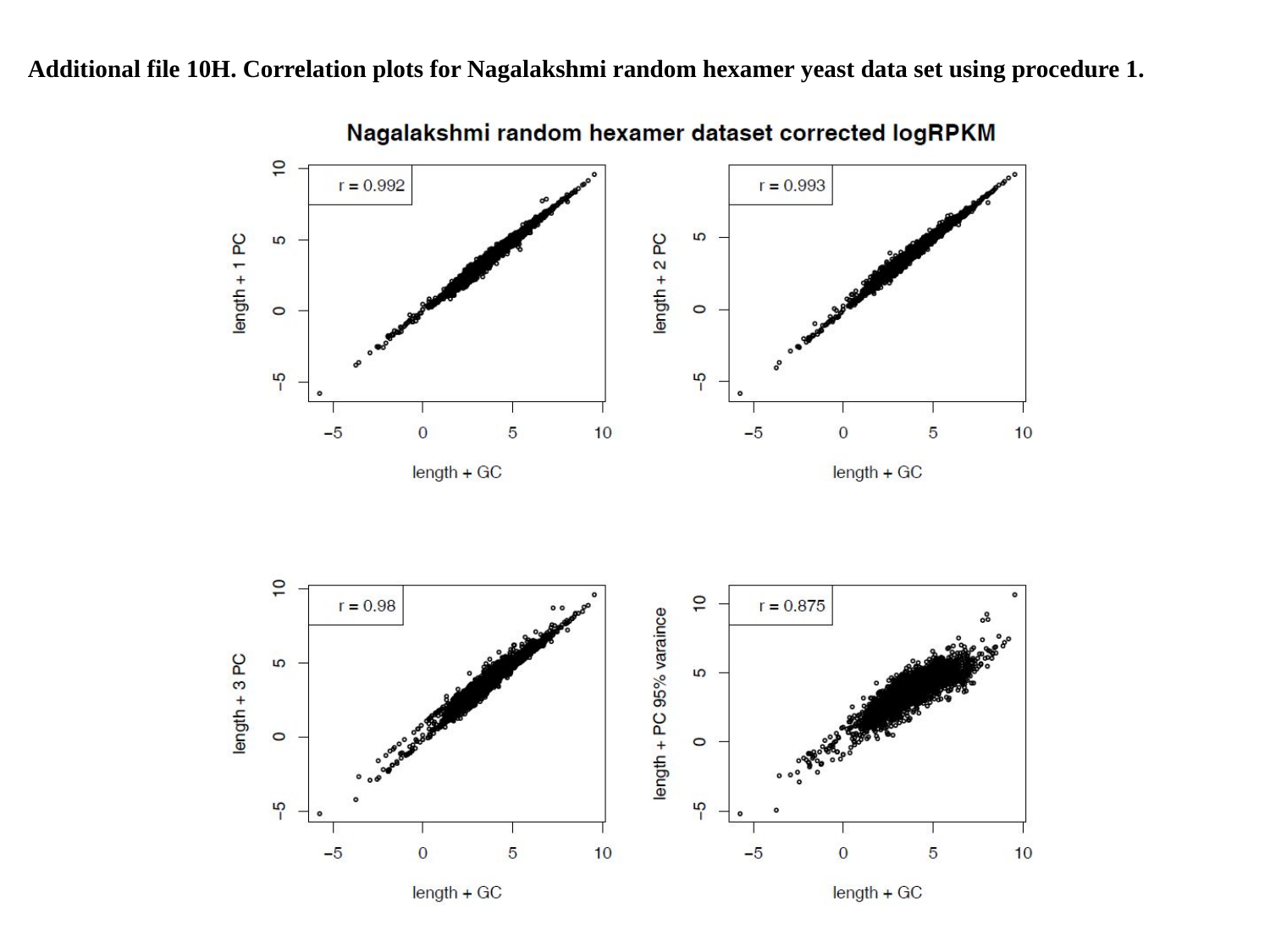

Additional file 10H. Correlation plots for Nagalakshmi random hexamer yeast data set using procedure 1.
