# Supplementary material for: Bias detection and correction in RNA-Sequencing data
Source: BMC Bioinformatics. 2011 Jul 19;12:290. doi: 10.1186/1471-2105-12-290 (PMC3149584; doi:10.1186/1471-2105-12-290)
Supplement: Additional file 11 — Plots of the component smooth functions that make up the fitted GAM objects with two predictors: gene length and GC content. The left panel shows the 1-D smooth for log gene length, and the right panel shows the 1-D smooth for GC content. The rug plot at the bottom of each panel shows the values of each predictor. The dotted lines are 2-standard errors above and below the estimated smooth (solid lines). Subfigures A-H are plots for 8 data sets in the same order as in Additional file 5. [file 1471-2105-12-290-S11.PPT]

## Slide 1
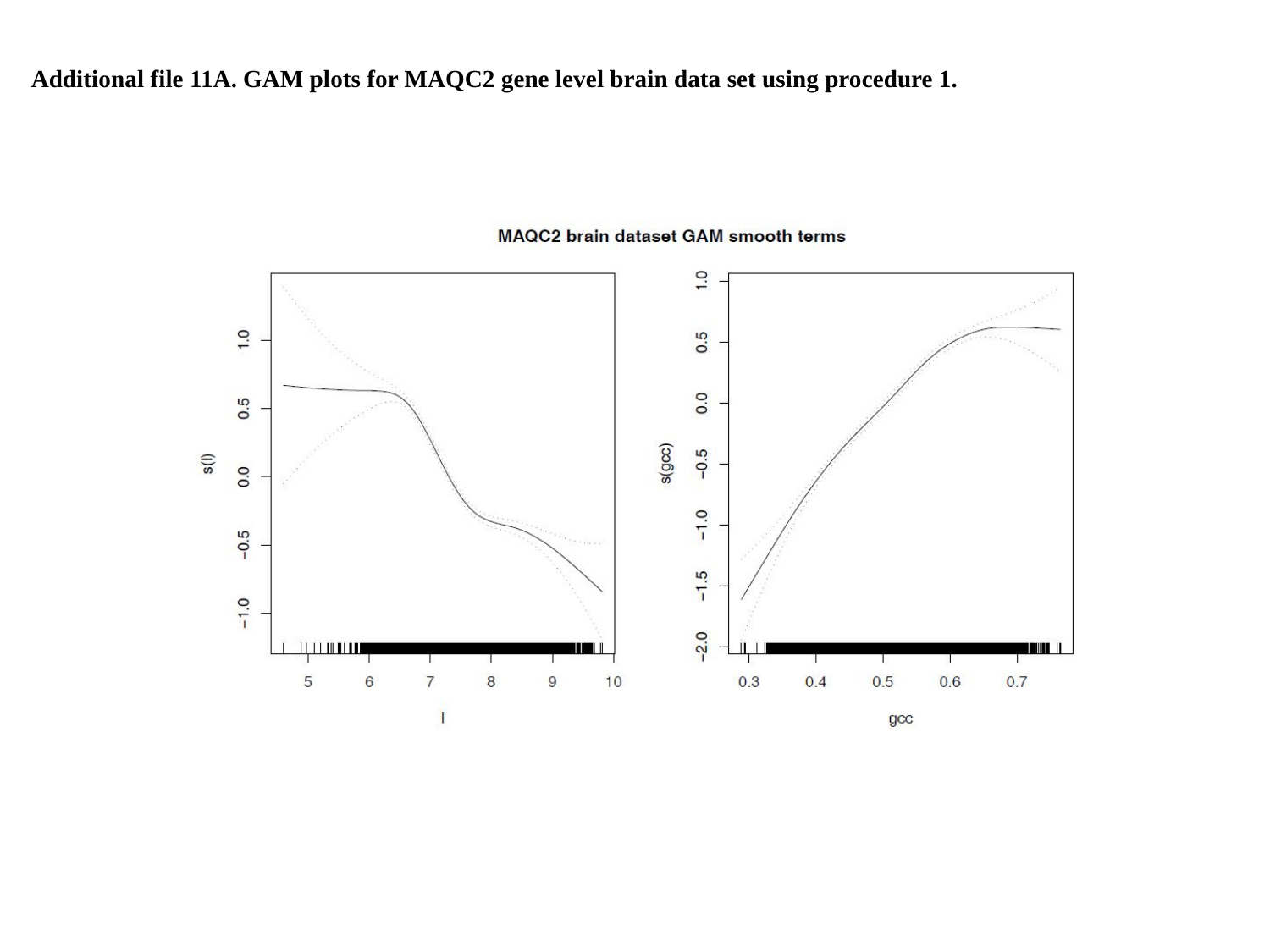

Additional file 11A. GAM plots for MAQC2 gene level brain data set using procedure 1.

## Slide 2
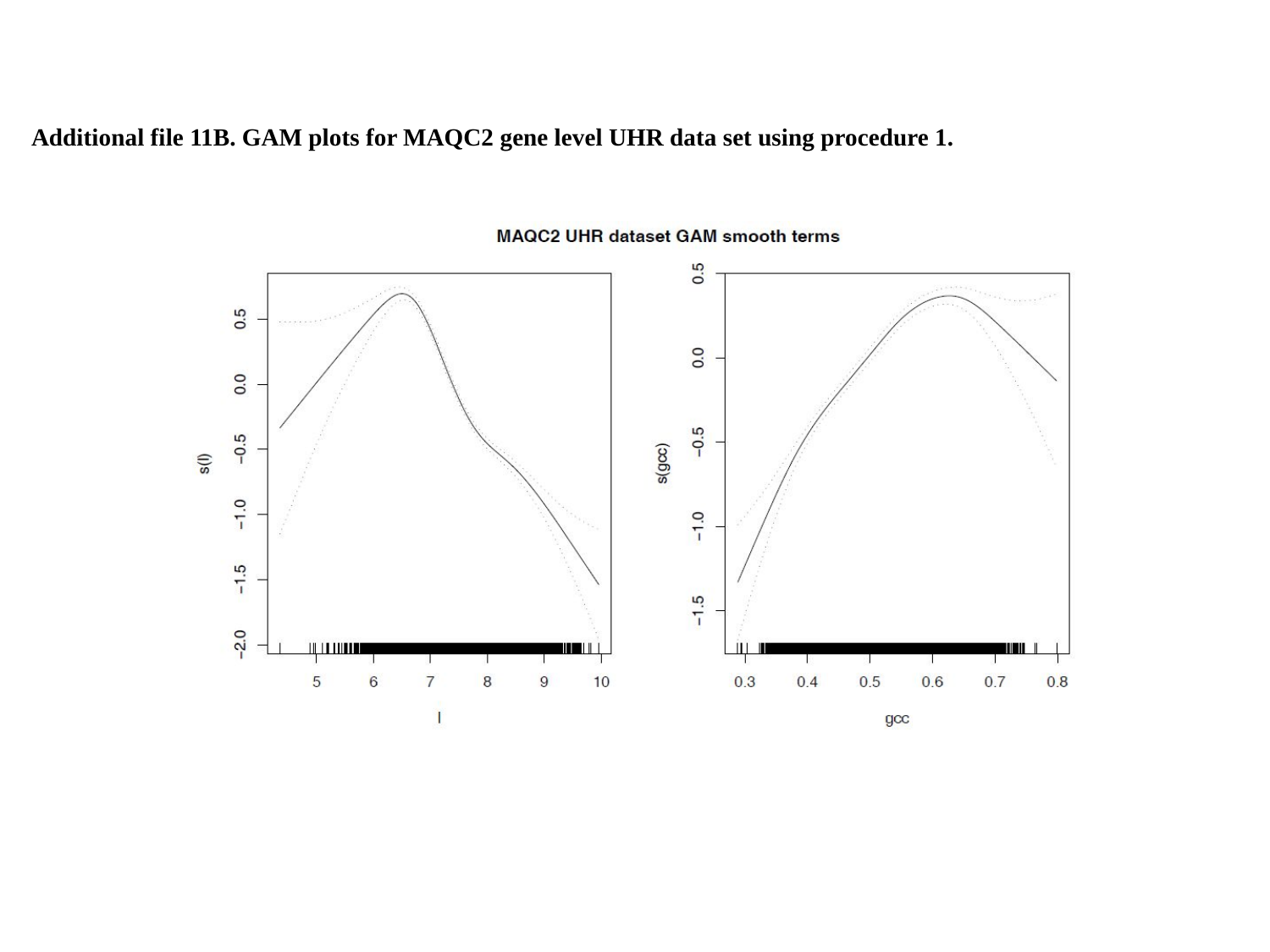

Additional file 11B. GAM plots for MAQC2 gene level UHR data set using procedure 1.

## Slide 3
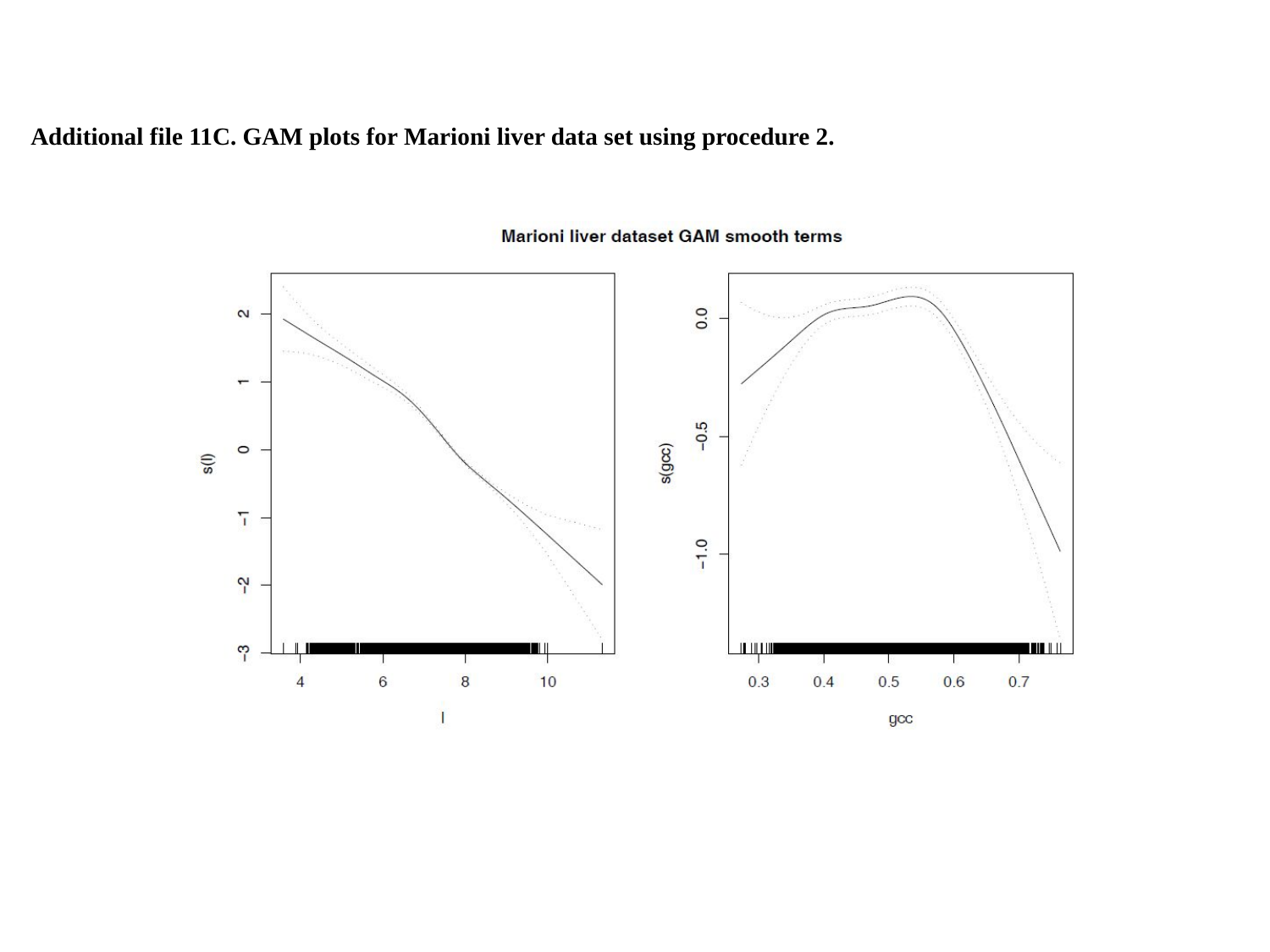

Additional file 11C. GAM plots for Marioni liver data set using procedure 2.

## Slide 4
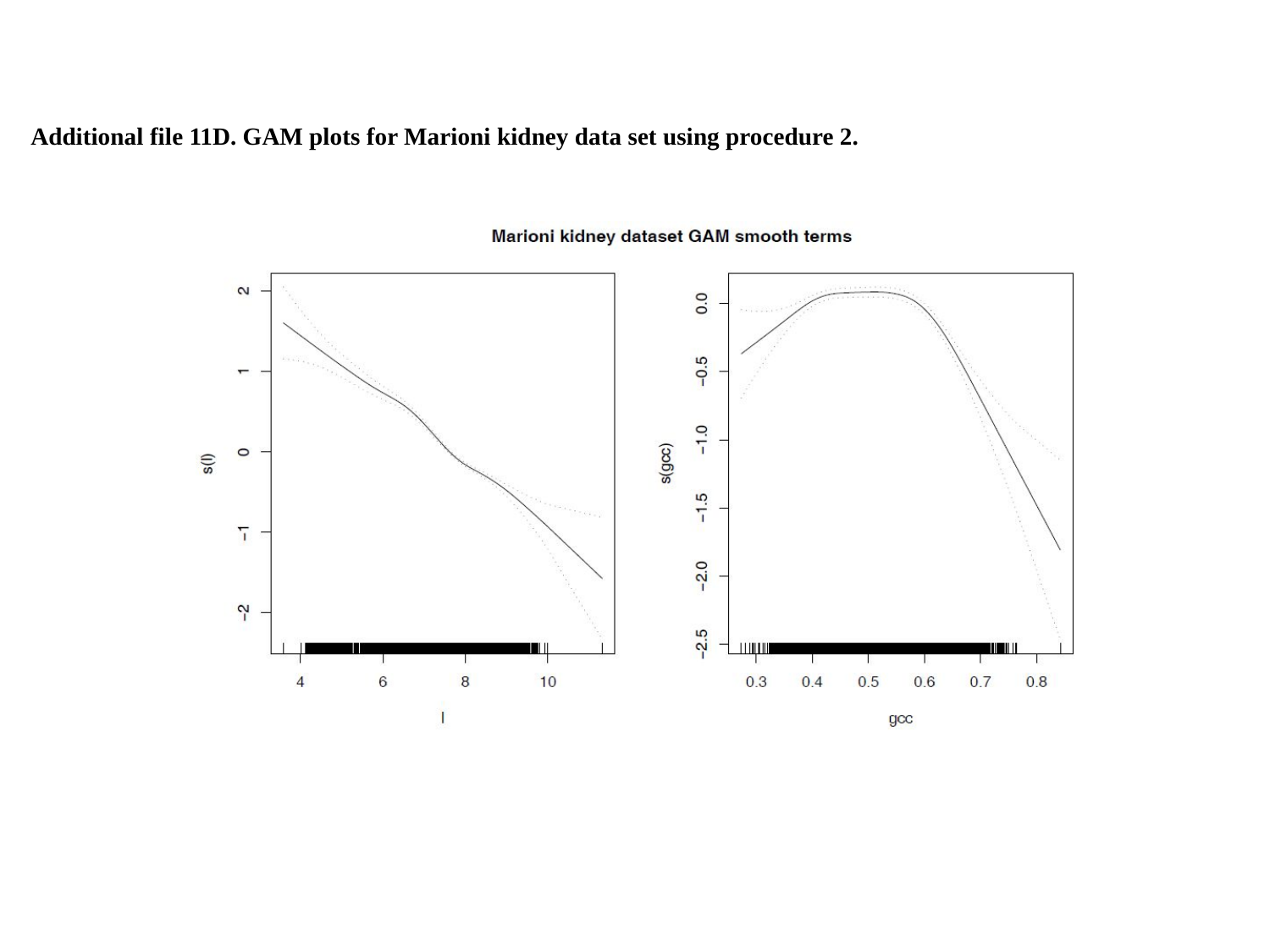

Additional file 11D. GAM plots for Marioni kidney data set using procedure 2.

## Slide 5
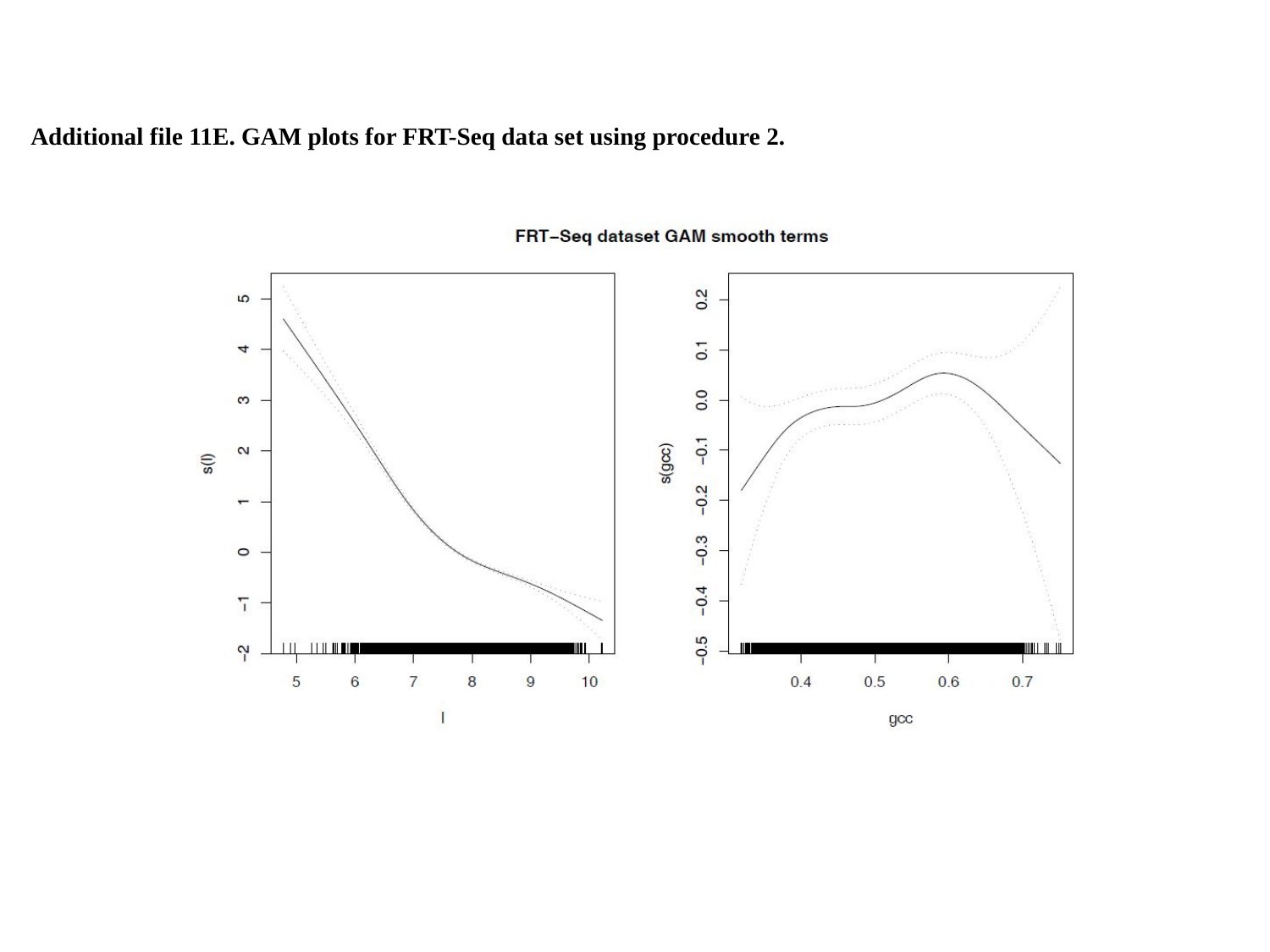

Additional file 11E. GAM plots for FRT-Seq data set using procedure 2.

## Slide 6
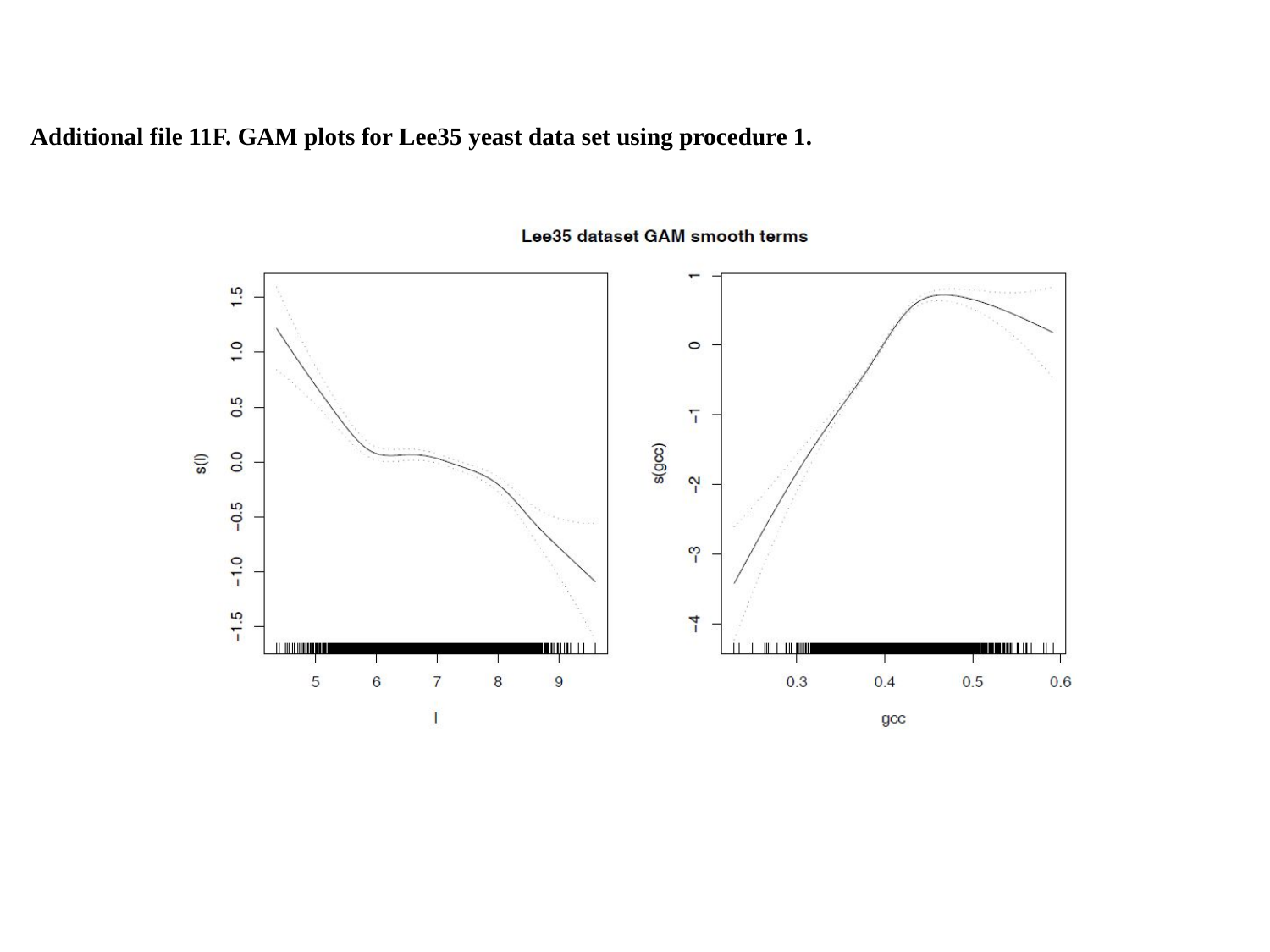

Additional file 11F. GAM plots for Lee35 yeast data set using procedure 1.

## Slide 7
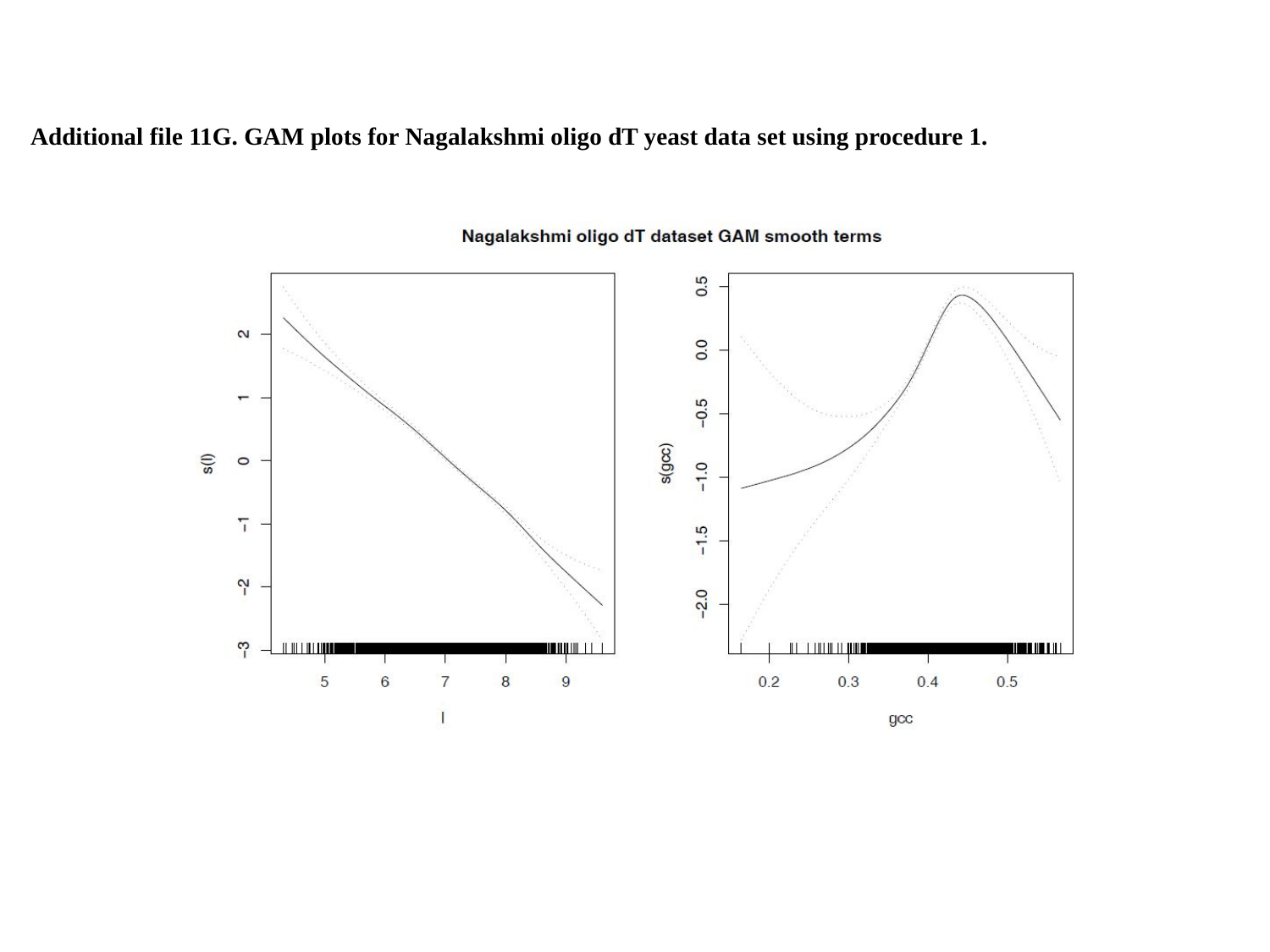

Additional file 11G. GAM plots for Nagalakshmi oligo dT yeast data set using procedure 1.

## Slide 8
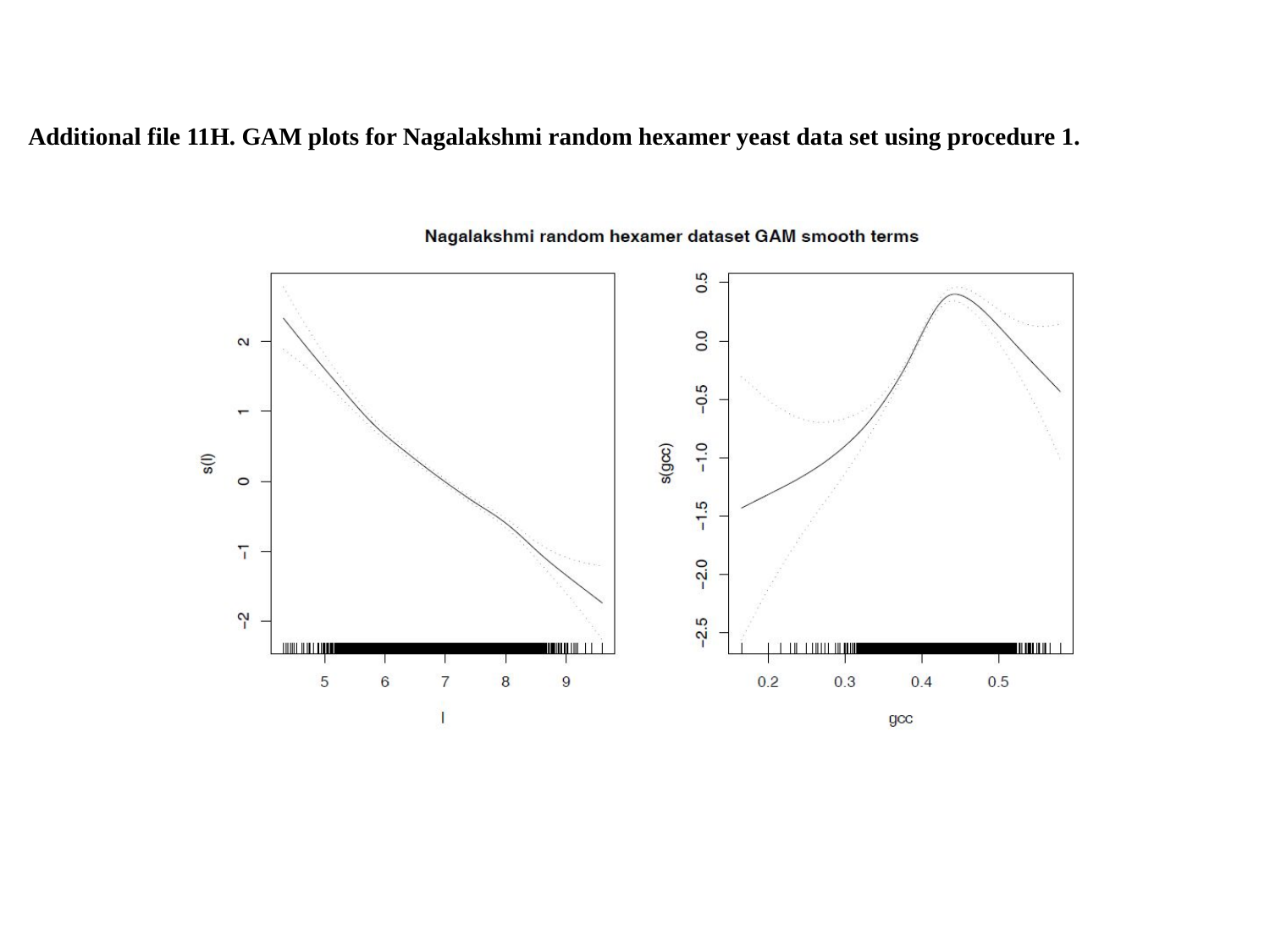

Additional file 11H. GAM plots for Nagalakshmi random hexamer yeast data set using procedure 1.
