# Supplementary material for: Bias detection and correction in RNA-Sequencing data
Source: BMC Bioinformatics. 2011 Jul 19;12:290. doi: 10.1186/1471-2105-12-290 (PMC3149584; doi:10.1186/1471-2105-12-290)
Supplement: Additional file 12 — Bias plots for MAQC data using Procedure 3 and comparing with random hexamer bias correction method. [file 1471-2105-12-290-S12.PPT]

## Slide 1
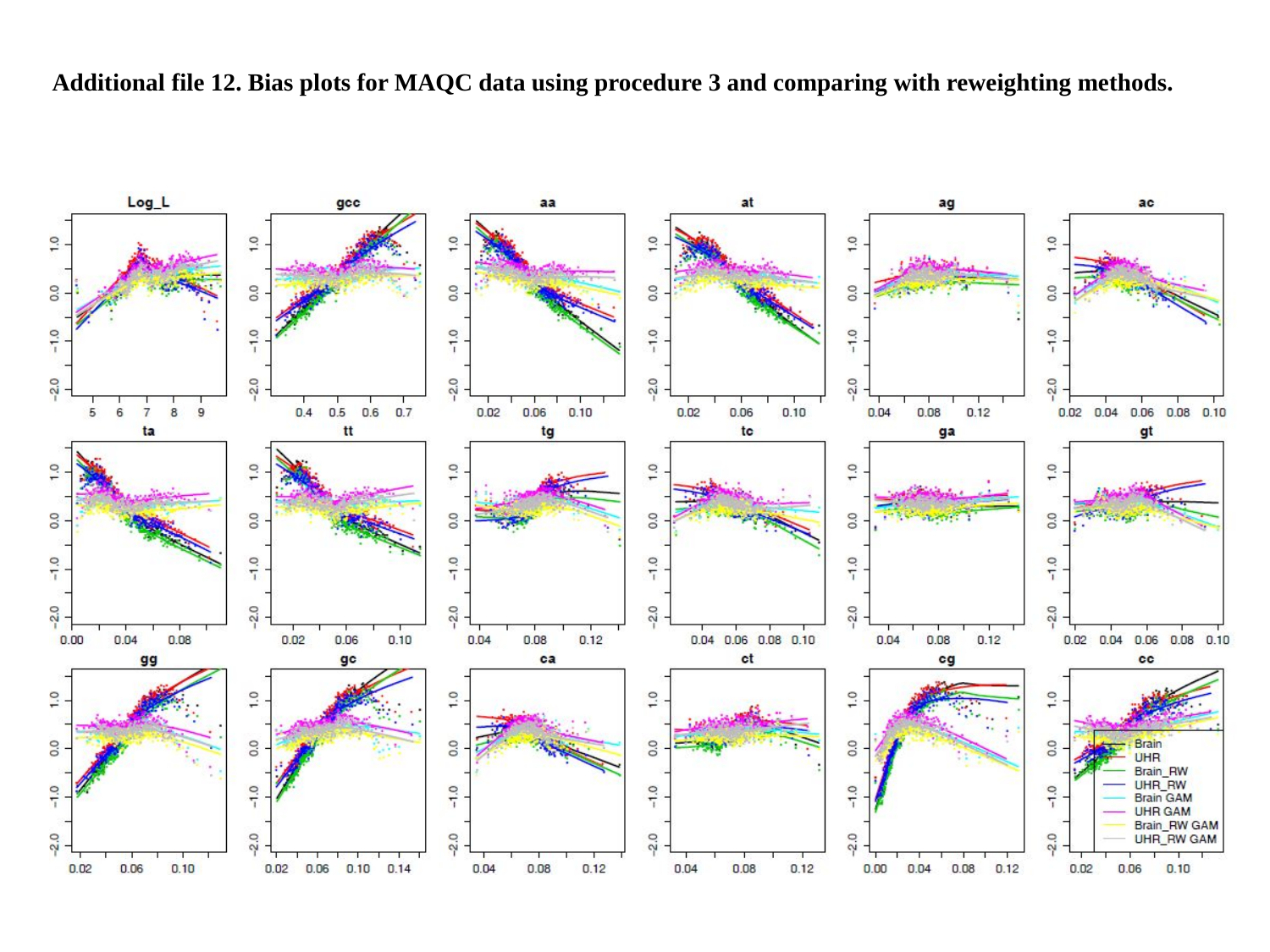

Additional file 12. Bias plots for MAQC data using procedure 3 and comparing with reweighting methods.
