# Supplementary material for: Bias detection and correction in RNA-Sequencing data
Source: BMC Bioinformatics. 2011 Jul 19;12:290. doi: 10.1186/1471-2105-12-290 (PMC3149584; doi:10.1186/1471-2105-12-290)
Supplement: Additional file 13 — Bias plots for SINO genes in Lee data using Procedure 3 and comparing with random hexamer bias correction method and mseq method. [file 1471-2105-12-290-S13.PPT]

## Slide 1
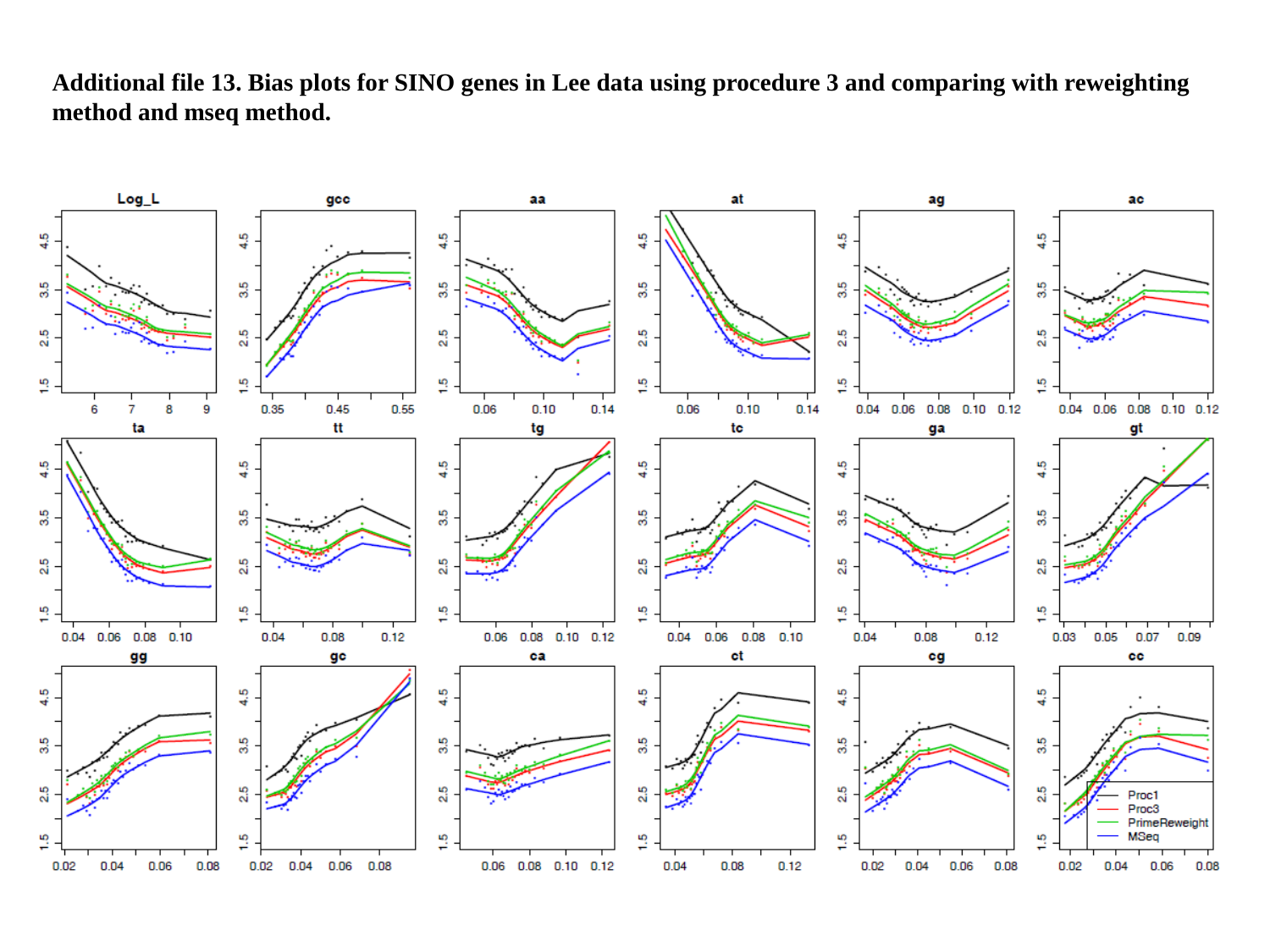

Additional file 13. Bias plots for SINO genes in Lee data using procedure 3 and comparing with reweighting method and mseq method.
